# Anatomy of a foreseeable disaster: Lessons from the 2023 dam-breaching flood in Derna, Libya

**DOI:** 10.1126/sciadv.adu2865

**Published:** 2025-03-28

**Authors:** Moshe Armon, Yuval Shmilovitz, Elad Dente

**Affiliations:** ^1^ Institute for Atmospheric and Climate Science, ETH Zurich, Zürich 8092, Switzerland.; ^2^ The Fredy and Nadine Herrman Institute of Earth Sciences, The Hebrew University of Jerusalem, Jerusalem 9190401, Israel.; ^3^ Cooperative Institute for Research in Environmental Sciences, The University of Colorado Boulder, Boulder, CO 80309, USA.; ^4^ School of Environmental Sciences, University of Haifa, Haifa 3103301, Israel.

## Abstract

Was the catastrophic flooding in Derna, Libya—one of the deadliest hydrometeorological disasters on record—an inevitable outcome of rare weather conditions, or did the design of the infrastructure fail to account for probable risks? On 10 to 11 September 2023, Storm Daniel, a Mediterranean tropical-like cyclone, caused heavy rainfall that led to the collapse of two dams and more than 5000 casualties in Derna. Using a combination of atmospheric reanalysis, satellite data, and hydrologic modeling, we overcame key limitations typical of data-scarce, high-variability regions and revealed that despite the catastrophic impact, the return periods of the rainfall and flood were only a few decades. Hydraulic simulations revealed that the dam failures amplified the damage nearly 20-fold compared to a dam-free scenario. With extensive and timely implications, our findings underscore the importance of uncertainty-aware risk assessment and highlight the value of distributed flood prevention and early warning systems in mitigating risks in vulnerable regions.

## Teaser

Dam failure, not extreme rainfall, exacerbated Libya’s flood disaster, exposing risks of over-reliance on flood protection.

## INTRODUCTION

Flash floods are the deadliest and costliest natural disaster in drylands [arid and semi-arid regions; (*1*, *2*)]. Therefore, flood attenuation and impact mitigation have always been a concern for dryland communities (*3*). Since the 1970s, the city of Derna, Libya, lying at the mouth of the ephemeral stream Wadi Derna, has profited from the construction of two major embankment dams upstream of the town (*4*, *5*). By mitigating floods, these dams presumably provided a sense of security that led to habitation and construction along the Derna channel and floodplain—ultimately amplifying the community’s vulnerability to extreme floods. Yet, extremely high precipitation variability characterizing drylands (*6*–*8*) often results in flood magnitudes much higher than anticipated (*9*–*11*). On 10 to 11 September 2023, an extreme flood flowed through Wadi Derna, destroyed both dams, and hit the city of Derna, resulting in more than 5000 casualties, with thousands more still missing (*12*), making it the deadliest hydrometeorological event worldwide at least since 2013 and one of the deadliest on record (*13*). Was this a black swan disaster that could not have been anticipated, i.e., an unknown unknown (*14*) or an event that should have been considered and is likely to occur under these circumstances?

Drylands are characterized by heavy-tailed precipitation and flood distributions, particularly for short (<24 hours) duration events (*9*, *15*, *16*), meaning that extremes tend to be more pronounced compared to frequent events (*11*). Short-duration heavy precipitation events in drylands, exacerbated by relatively small, convective-scale processes, often yield precipitation totals equivalent to the mean annual or even higher [e.g., (*17*, *18*)]. Runoff is typically discontinuous, generated only over certain parts of the catchment, where and when high-intensity precipitation meets bare, rocky surfaces (*19*). Therefore, to give stringent predictions of dryland floods, high spatiotemporal resolution meteorological, hydrological, and physiographic data are needed, and an event-scale analysis rather than a climatological perspective is often preferred (*20*).

However, both precipitation and streamflow data are generally scarce in drylands [e.g., (*21*, *22*)]. Consequently, determining the appropriate actions both for water resource management and for flood prevention and mitigation is particularly challenging, yielding both over- and underdesign of infrastructure. For example, the Mansour Eddabhi Dam in the arid Moroccan Atlas, designed for water management, remained empty for more than a dozen years after its construction because it was designed based on too short hydrological records (*8*). The design of dams for flood protection in drylands poses an even greater risk, as failure could result in immediate and tragic consequences.

**Fig. 1.**
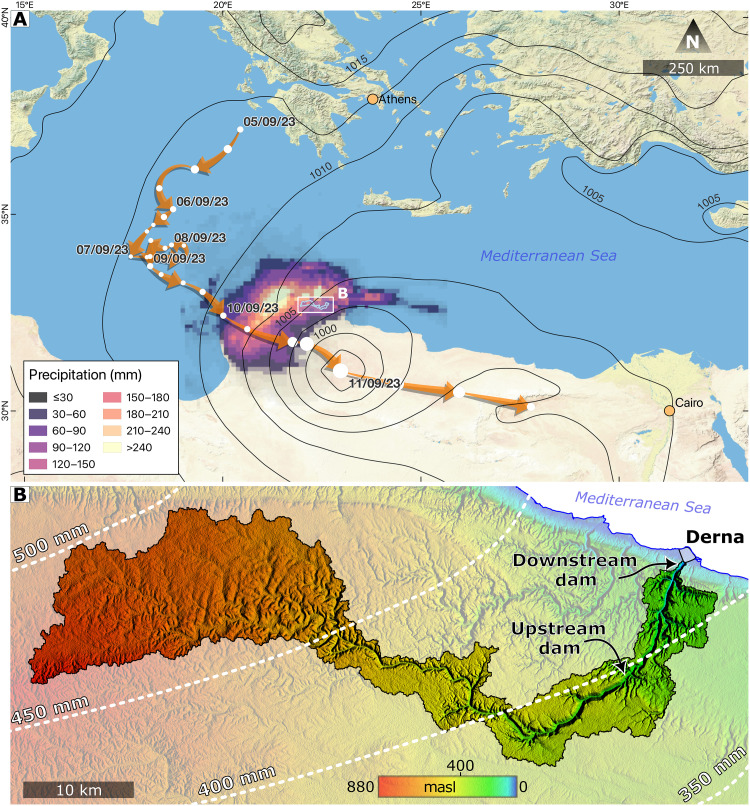
Storm Daniel’s path and the study region. (**A**) Path of the storm from 5 to 11 September and precipitation in northeastern Libya. White circles represent the centre of the storm in 6-hour intervals, and circle sizes indicate the storm’s central pressure (smallest circles = 1006 hPa, largest circle = 996 hPa). Cumulative daily precipitation in the storm’s vicinity during peak precipitation on 10 September 2023 is colored, the sea level pressure when the storm was deepest (11 September at 00:00 UTC) is in black contours, and the Wadi Derna catchment area is framed. (**B**) Wadi Derna catchment. Elevation in meters above sea level (masl) is colored [data from the Advanced Land Observing Satellite instrument: Phased Array type L-band Synthetic Aperture Radar (ALOS-PALSAR) (*61*)], and white contours denote mean annual precipitation [data from the Integrated Multi-satellitE Retrievals for the Global Precipitation Measurement mission (IMERG) (*51*)].

In Derna, the strategy chosen to protect the city from floods was the establishment of two embankment dams. These were built to prevent damage from flash floods in Wadi Derna, an ephemeral stream that drains 575 km^2^ at its outlet to the Mediterranean Sea (Fig. 1B), flowing only as a consequence of heavy precipitation events. The upstream larger dam of the two in Wadi Derna drains the wetter part of the basin (453 km^2^), with a storage capacity of ∼22 × 10^6^ m^3^ (fig. S1), roughly 15 times larger than the downstream dam capacity. Stream monitoring data in the Derna catchment, if any exist, are not publicly available, impeding the ability to assess the potential magnitude of extreme events. A few studies used rainfall-runoff models to estimate extreme flood magnitudes (*5*) of different return periods (*23*), resulting in values much greater than the dam capacities, implying a major design shortcoming. Because embankment dams are typically not intended to allow overflow of water, floods exceeding their designed maximum storage capacity puts these dams in severe danger of failure (*24*). Before the storm Daniel flood, both dams were reported to suffer from damage caused by previous floods and were thus in an exacerbated danger of collapse (*5*). Moreover, the complicated political, social, and economic situation in Libya since 2011 (*25*) prevented centralized actions to ensure both dam maintenance and effective risk communication to the downstream community.

The devastating Storm Daniel flood embodied the dam collapse threat. However, the reasons for the extreme destruction by the flood are still undetermined. Were the storm and the flood so extreme that the damage was inevitable? What is the role of the dam bursts in the tragic outcome; how destructive was the flood if the dams had not been constructed? And, what can be inferred from this flood to improve risk management and mitigation strategies elsewhere? To answer these questions, we investigated and simulated the chain of events that led to this disaster: from the atmospheric disturbance and the resulting rainfall to the basin’s hydrological response to the filling and collapse of the dams and the consequential destruction in the city of Derna.

## RESULTS

### The torrential rains of storm Daniel

The Mediterranean tropical-like storm Daniel formed over the Ionian Sea in early September 2023, when Mediterranean Sea surface temperatures peak. The storm triggered heavy rainfall and floods in large parts of Greece, western Turkey, and parts of Bulgaria, with substantial property damage and more than two dozen casualties (*26*). From 5 to 9 September, the storm wandered slowly southward (Fig. 1A). It then deflected eastward and made landfall on 10 September, passing over the Cyrenaica region (eastern Libya) into the northern Sahara and dissipating slowly in the southeastern Mediterranean region. Occasionally, increased sea surface fluxes and latent heating from cloud condensation intensify preexisting Mediterranean cyclones and turn them into tropical-like storms, and once or twice a year, on average, somewhere in the Mediterranean, hurricane-strong storms are developed (*27*). These storms, termed medicanes, have an increased destructive potential due to high winds and heavy precipitation (*28*). The warm-core storm Daniel produced winds of nearly 30 m s^−1^ (fig. S2) and could therefore be considered a medicane.

Mediterranean cyclones in Cyrenaica rarely occur before mid-October and are exceptional in early September (fig. S3). When cyclones do occur in the area, sea level pressure seldom drops below 996 hPa (*29*, *30*). Using reanalysis data going back to 1950, we determined the rarity of these storms. Based on 6-hour resolution cyclone detection (*31*), 1339 cyclone centers have been observed in the vicinity of Derna since 1950 (1.25% of the total time). During 22 of these cyclones (1.69% of the cyclones), pressure had dropped below the minimum pressure exhibited during the storm Daniel (fig. S4). Empirically, this translates into a return period of ∼3 times per decade. Storm Daniel exhibited anomalies of roughly three to four SDs not only in terms of sea level pressure but also mid-troposphere geopotential height and specific humidity in the lower troposphere (fig. S5). The storm was unique because it struck the North African coastline very early in the rainy season when sea surface temperatures peak (*32*), whereas all the other cyclones with a minimum pressure lower than storm Daniel occurred between January and May (fig. S4).

Calibrated precipitation in northeastern Libya during the storm’s passage (Fig. 1A and Materials and Methods) was very high compared to normal September days, accumulating to nearly a third of the mean annual precipitation (Fig. 1B). Whereas ground reports in Cyrenaica outside of the Derna catchment showed precipitation values of up to 414 mm day^−1^ at the peak of the event (fig. S7), over the catchment, precipitation was less than ∼200 mm day^−1^ (Fig. 1). This value is higher than any other daily value since satellite-based precipitation data exist (June 2000; fig. S8). However, when converted into return periods, daily precipitation intensity over the catchment exhibited values of only a few tens of years; its maximum is <80 years based on the simplified metastatistical extreme value (SMEV) method (*33*), which is especially suitable for drylands (figs. S9 to S11, and Materials and Methods). This means that precipitation during the storm was intense, but not to the level that might be inferred solely from the tragic result.

### The hydrological response of Wadi Derna to the storm

**Fig. 2.**
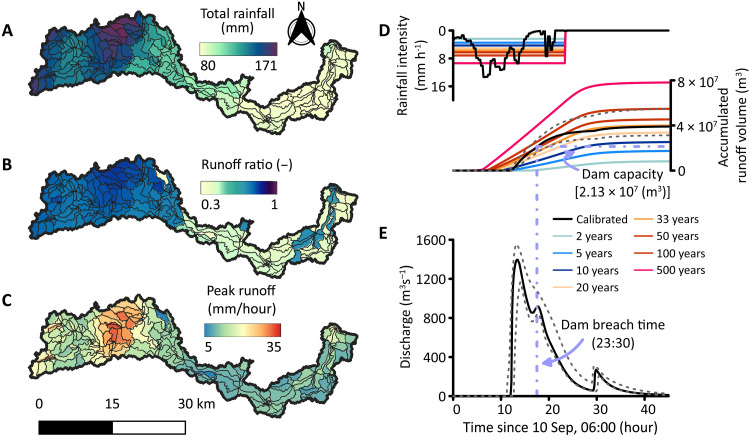
The rainfall-runoff model results. (**A**) Storm Daniel’s total rainfall over the Wadi Derna watershed. Black polygons denote the hillslope units used for runoff modeling (Materials and Methods). (**B**) Hillslope runoff ratio (runoff at the hillslope outlet divided by precipitation on the polygon area). (**C**) Hillslope peak runoff rate. (**D**) Basin-average rain intensity (left axis) and calibrated flood volume at the upstream dam (Fig. 1B) during storm Daniel (right axis) are denoted by black lines. Rain intensities and flood volumes corresponding to various return periods are denoted by colored lines. (**E**) Simulated Derna flood hydrograph. The uncertainty range is based on simulated hydrographs with relative bias scores up to 5% higher (worse) than the best score (see the Materials and Methods). Vertical and horizontal purple dashed lines mark the modeled dam breach time and the dam capacity (23:30 UTC, 21.35 × 10^6^ m^3^), respectively.

Hydrological simulations reveal that the return period of the flood is also on the order of a few decades. We calibrated a rainfall-runoff model based on the capacity of the upstream dam (fig. S1) and the estimated timing of its collapse (relative bias error <1%, which translates into uncertainty range in Fig. 2, fig. S14, and Materials and Methods). Results of these simulations show the flood’s peak discharge at the upstream dam site was *Q* ≈ 1400 (*Q*_max_ = 1550, *Q*_min_ = 1160) m^3^ s^−1^ (Fig. 2), placing it slightly below, and almost three times lower than the southeastern and western Mediterranean envelope curves, respectively (*34*, *35*). While these envelope curves are not from Cyrenaica itself, they are, to the best of our knowledge, the closest available and provide a reference for assessing the relative magnitude of the event. To evaluate the recurrence frequency of the flood, we compared the flood’s accumulated volume, *V* = 39 (*V*_max_ = 55, *V*_min_ = 31) × 10^6^ m^3^, with hydrological simulations of various return period synthetic storms using pixel-based estimates of the daily rainfall intensity as input (Fig. 2D and Materials and Methods). This comparison reveals that the return period of the flood is 30 to 50 years.

While the entire basin contributed runoff to the flood, its upper part generated the bulk of the volume (Fig. 2B). There, higher precipitation rates hit fine-texture low-conductivity soils (Fig. 2A and fig. S12B), resulting in very high runoff values (Fig. 2, B and C). The runoff ratio at the upper part of the basin approached 70%, which is very high for these environments where watershed-scale runoff ratios typically range from a few to tens of percents (*36*, *37*). At the lower parts of the basin, high runoff ratios only appeared over spatially limited rocky hillslopes, adjacent to the main stream (fig. S12). The high portion of runoff contributed from the upper part of the basin, >30 km of the upstream dam, is manifested in the 4-hour lag between the peaks of rainfall intensity and flood discharge (Fig. 2E); while maximum rain intensity was in the late afternoon, peak discharge was in the evening of 10 September. Three hours after the flood peak at the upstream dam, cumulative flood volume surpassed the dam capacity. With only a small bell-mouth spillway, capable of discharging water at a rate seven times lower than the flood discharge (figs. S15 to S17), the dam was doomed to be rapidly overtopped.

### Role of the dam collapse

**Fig. 3.**
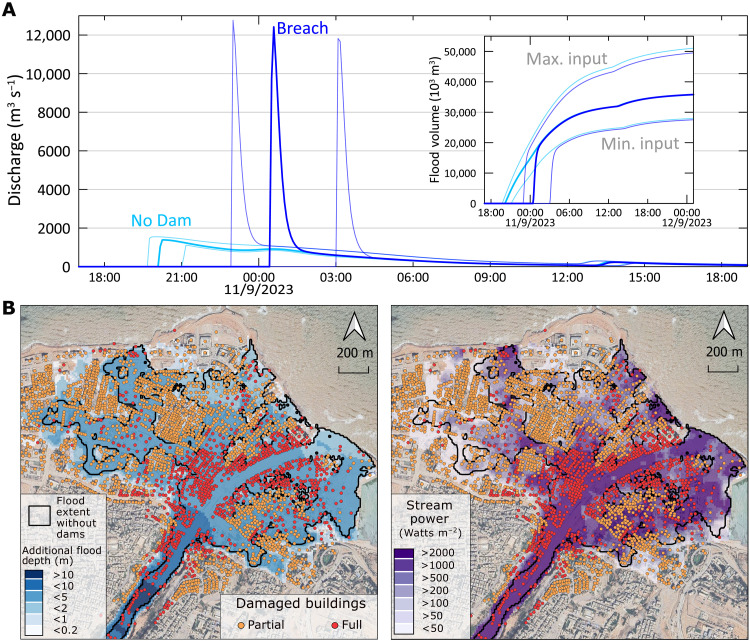
Simulated discharge, depth, and SP under the dam breach and no-dams scenarios. (**A**) Hydrograph and cumulative volume of the floods, 0.5 km upstream of Derna. Thin lines denote the uncertainty range based on the hydrologic model output (Fig. 2E). (**B**) Maximum depth differences between the two scenarios (left) and the SP simulated for the breach scenario. In most of the flooded area, the SP of the breach flood is 2 to 100 times larger than the SP of the no-dams flood (fig. S22). Also shown are post-flood imagery from Google (Maxar Technologies, 13 September 2023) and a classification of the damaged buildings in the city from the UN-Habitat (*39*).

To quantitatively evaluate the dam collapse effect, we used the rainfall-runoff model output as input to hydraulic simulations of two different scenarios: a realistic dam breaching scenario and a counterfactual scenario in which there are no dams in the Wadi. The dam-breach flood simulation yielded a peak discharge of ∼12,400 m^3^ s^−1^ at the outlet of Wadi Derna, eight times higher than the peak of the simulated flood without dams in the wadi (Fig. 3A). The two flood scenarios differ in the shape and timing of their hydrographs too: The dam breach hydrograph peak is delayed by 4 hours versus the no-dams scenario and its shape is much sharper. Within the extent of Derna City, the maximum flooded area is 67% larger in the breach compared to the no-dams scenario (Fig. 3B). Moreover, the breach scenario exhibits much higher flood depths, with most of the inundated area showing 2- to 5-m deeper water at the maximum stage (fig. S19).

The rate of energy expenditure by the stream flow per unit of bed area, known as flood unit stream power [hereafter referred to as stream power (SP); (*38*)], defined as the product of the bed shear stress and the flood’s mean velocity, is orders of magnitude higher in the dam breach scenario (figs. S19 and S22). Where the flooded areas of the two scenarios overlap, the breach scenario shows 10 to 100 times higher SP values than those in the no-dams scenario. This substantial difference between the two scenarios reveals that much of the damage is associated solely with the breach of the dam.

**Fig. 4.**
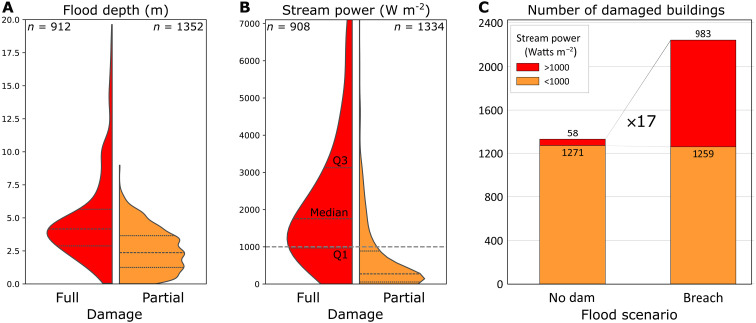
Damaged buildings by flood depth and SP. (**A**) Distributions of the simulated maximum flood depth (breach scenario), classified into buildings that were fully and partially damaged in the Derna flood (Fig. 3B). (**B**) Distributions of flood SP [presented similarly to panel (A)]. (**C**) The number of buildings affected by the flood under the scenarios of no dams and dam breach, classified by SP of 1000 W m^−2^ as a threshold for substantial damage (see also fig. S20).

A short time after the disaster, damage to buildings in Derna City was mapped through satellite imagery [Fig. 3B and (*39*)]. This mapping further emphasizes the substantial differences between the impact of the “natural” no-dams flood and the catastrophic flood that resulted from the dam breach. According to the damage mapping, the storm Daniel flood damaged 3087 buildings, of which 1015 were fully destroyed. In the breach simulation, 2271 buildings, 78% of the documented damaged buildings downstream Wadi Derna, were affected by the flood. Most of the documented damaged buildings not affected by the simulated flood are located on the banks of Wadi Derna; these buildings were presumably affected by floods from adjacent watersheds or bank erosion, which were not simulated in our model. Other differences between the extent of the damaged buildings and the simulated flood are attributed to the digital elevation model’s (DEM) resolution, as reflected by the nonflooded neighborhoods in north-west Derna (Fig. 3B). Of the fully damaged buildings, 912 (93%) were affected by the simulated dam breach flood. Most of these buildings experienced maximum flood depths >3 m, whereas partial damage was documented mostly in areas experiencing shallower flood depths (Fig. 4A).

The maximum SP exhibited during the dam breach flood simulation is a good estimator for the damage caused by the flood and shows that the damage associated with the dam breach is substantially larger than the potential damage in the no-dams scenario. While both flood depth and SP are significantly higher (one-sided Mann-Whitney *U* test; *P* < 0.001) in the group of fully damaged buildings compared to the group of partially damaged buildings (Fig. 4, A and B), SP is a much better discriminator (fig. S20). Most of the fully damaged buildings were affected by a maximum SP >1000 W m^−2^, which is higher than the in-channel 300 W m^−2^ used as a threshold for “major channel morphological adjustments” (*40*). In Derna, some destroyed buildings are located far from the main stream channel, without the apparent impact of channel bed erosion (*41*), which may explain the much higher energy flux needed to directly damage infrastructure in these Derna neighborhoods.

Using this 1000 W m^−2^ threshold for substantial damage, we evaluate the difference between the breach and no-dams’ scenarios in their destruction potential (Fig. 4C). Approximately 40% of the flooded area in the breach scenario exhibits threshold-exceeding SP values compared to <5% observed in the no-dam scenario (fig. S21). While the number of buildings affected by SP values lower than the damage threshold is similar in the two scenarios, the number of fully damaged buildings (i.e., values higher than the damage threshold) is almost 20 times higher in the dam breach scenario, demonstrating amplified impact caused by the dam breach.

## DISCUSSION

### Reducing uncertainty in flood prediction for drylands

Through an interdisciplinary methodological framework based on globally available data, we examine the return periods of storm Daniel’s rainfall and flood, and the flood damages. Our results indicate that storm Daniel’s flood in Derna was exceptional in terms of damage, albeit not that extreme in terms of the natural processes leading to the flood. On the basis of the atmospheric reanalysis data, we estimate the frequency of extreme weather events for the past >70 years and show that cyclones as deep as storm Daniel are not very rare (∼3 times per decade). However, the early-in-season arrival of the cyclone to Cyrenaica’s coastline presumably affected the rainfall yield of the cyclone and led to the most extreme rainfall in the area since the early 2000’s. Our approach, which narrows the uncertainty where records are short and rain and floods are characterized by high variability, reveals that the observed daily rainfall is expected once every several decades (<80 years). Further, by combining calibrated remote-sensing precipitation with hydrological modeling, we constrained the flood volume return period to 30 to 50 years and showed that its peak discharge is lower than the envelope curves of Mediterranean regions. Such a storm is exactly the kind a dam is expected to protect from.

The construction of the dams upstream of the city of Derna resulted in two unfortunate consequences: (i) The collapse of the upstream dam is the main cause for damage within the city (Fig. 4C), and (ii) the dams deceptively enabled residing in the channel and its floodplain. Through hydraulic simulations, we showed that without the dams, flood discharge and the inundated area (Fig. 3) would have been much smaller, and damages substantially reduced. Combining these tools, our approach provides important information for watershed and risk management that can be useful in other environments, especially in drylands, where flood risk estimates are too uncertain and where drainage basins are often ungauged.

Reliable, long-term observations are key in providing robust flood return period estimates, which are cornerstones in planning flood prevention measures. These estimates are subject to high uncertainties, predispositions, and variability when using different datasets. For example, Ashoor and Eladawy (*42*) reported large variability for the 1-in-1000–year flood volume in Wadi Derna; this volume was estimated to be 14 × 10^6^ m^3^ in the 1970s, when the dams were designed, but reevaluated to 67 × 10^6^ m^3^ in 2003. Given that storm Daniel flood yielded 39 × 10^6^ m^3^, in the 1970s it would have been considered as a most extreme, improbable event, with a return period exceeding 1-in-1000 years, whereas in the 2000s, it would have been estimated as a much more frequent event (Fig. 2D). Storm Daniel’s flood volume, which overtopped the dam and far exceeded its design capacity, implies that the dam failure resulted from inadequate design (*24*) rooted in inherent uncertainties in flood volume estimation, rather than a mechanical malfunction.

In addition to the inherent data and methodological challenges, climate change introduces another layer of complexity to return period estimation. On the basis of the precipitation data from reanalysis, climate change has been suggested (*43*) to increase the likelihood of rainfall, such as produced by storm Daniel in the vicinity of Derna, by a factor of three—shifting it from a 1-in-2000–year to a 1-in-600–year event. These estimates are much larger compared to what we show here based on calibrated remote-sensing rainfall data. However, the uncertainty reported for this factor ranges widely from 0.5 to 2100. While climate change accounts for a possible increase in the intensity of extreme rainfall in Derna and other drylands, the very high precipitation variability, expressed by the huge uncertainty range, challenges the detection of climate change signals (*43*, *44*).

### Lessons from Derna: A call for action

The impact of Storm Daniel’s moderately high-magnitude rainfall was heavily exacerbated by the failure of the dams in Wadi Derna; jointly, it produced one of the most devastating natural disasters in the 21st century. The Derna disaster should serve as an alarming sign that frequency analyses of extreme events in drylands should take into account the very high natural climate variability, the different and evolving approaches to derive extreme value estimates, changes in land use and runoff coefficients, and climate change impacts. These challenges cast doubt on the economic and the environmental rationale for dam construction as a strategy to mitigate flood risks in drylands.

The disaster in Derna underscores the urgent need for adopting suitable statistical methods and improved representation of climate change to better constrain return period estimates in drylands. While novel statistical techniques, such as SMEV, should be adopted, it is important to acknowledge that statistical methods are continually evolving—estimates may change, and today’s uncertainties may be better resolved by the time these analyses become critical. Yet, it is perhaps even more important to adopt uncertainty-aware approaches that incorporate the inherent unknowns into resilient flood prevention strategies, ensuring that the risk of failure remains low enough to be acceptable. To minimize uncertainties, another strategy is to leverage paleoflood hydrology practices, valuable for quantifying past floods and shedding light on the potential magnitude of future floods. This approach is particularly important in regions where hydrometric data are scarce and have proven effective in constraining the magnitude of extreme events (*45*).

To address the uncertainties and risks identified above, future flood mitigation measures should prioritize distributed nature-based solutions [e.g., (*46*, *47*)] and adopting flood warning systems as integral components alongside prevention strategies (*48*, *49*). Crucially, the absence of publicly available rain and stream gauge data, as well as return period estimates used for the design of the dams, represents a fundamental failure in disaster preparedness. These data must be made accessible to promote transparency, enable critical evaluation, and drive innovative solutions. These measures are not merely recommendations; they are imperatives if we are to avoid repeating the catastrophic failures witnessed in Derna.

The lessons from Derna are not confined to this region; similar risks are present globally, requiring immediate attention from both the scientific community and policymakers. Our results strongly imply that the complete failure of other dams is not a matter of if, but rather when. We urge the scientific community, local knowledge holders, and authorities to collaborate on this timely hazard to improve knowledge, data availability, and risk communication and to re-evaluate the planning of habitat areas along dryland streams to prevent the next disaster.

## MATERIALS AND METHODS

### Meteorological data processing

Meteorological data were obtained from the European Centre for Medium-Range Weather Forecasts (ECMWF) Reanalysis v5 (ERA5) reanalysis (*50*). Anomalies in geopotential height, sea level pressure, and specific humidity were computed by comparing these variables during the cyclone’s most intense phase (lowest sea level pressure; 11 September 2023, at 00:00 UTC) with the climatology of the same fields for all the days in September during 1979–2023 at the same time in the day.

Cyclone frequency and statistics were derived from binary cyclone masks based on ERA5 sea level pressure maps during 1950–2023, in which cyclones are defined as enclosed regions containing one or more sea level pressure minima (*31*). Cyclone centers are defined as the grid points with the lowest pressure within a cyclone.

Wind speed over the Mediterranean during the passage of storm Daniel was obtained from the Meteorological operational satellite C Advanced Scatterometer (MetOp-C ASCAT) Level 2 Ocean Surface Wind Vectors Optimized for Coastal Ocean product through the High-Level Tool for Interactive Data Extraction (HiTIDE V 4.10.0) at https://hitide.podaac.earthdatacloud.nasa.gov/.

### Precipitation data and calibration

We used precipitation data from the Integrated Multi-satellitE Retrievals for the Global Precipitation Measurement mission (IMERG; *51*) at half hourly intervals and 0.1° in space. IMERG is considered one of the most reliable globally available rain products for quantitative precipitation estimations in drylands [e.g., (*22*)]. Sources for precipitation estimation inaccuracies include (i) the local intensity errors, such as below-cloud rain evaporation, yielding rainfall overestimation (*52*) and (ii) a spatial error resulting from the spatial shifting of the local intensity values according to motion vectors derived from global reanalysis (*53*). While intensity bias can be minimized by calibrating precipitation estimations to in situ observations, the spatial shift error can be minimized by making spatial adjustments such as morphing (*54*) and shifting (*18*) of the rain local intensity values.

To obtain an optimal estimation of rainfall during the event, we calibrated nongauge-corrected IMERG-late V06 data (*51*) with daily gauge data published by the Libyan National Center for Meteorology on social media (fig. S7 and table S1). Because of the different potential error sources, the calibration process consisted of two steps: (a) spatial matching and (b) bias removal. In step (a), we searched within a 5 × 5 window (±2 pixels at the zonal and meridional directions), the spatial shift that minimizes both the root mean square error and bias (fig. S6). The best overall value was found when shifting the rain field one pixel step to the east and two to the south. In step (b) of the calibration, we adjusted the remaining mean field bias.

### Rainfall return period

To estimate the extremeness of precipitation during the storm, we computed the return periods for daily intensity values based on the SMEV method (*33*) using IMERG-final V06 data spanning June 2000 to May 2021. The method is especially valuable in regions characterized by a low number of rain days and when records are short and was already tested and used in and next to the study region (*22*, *55*). SMEV requires defining a few parameters that we set similarly to (*22*): (i) a threshold for rain days, which is here defined as 1 mm day^−1^; (ii) a percentile defining the stretched exponential right tail, defined here as the upper 25% of rain days; and (c) a temporal separation between “ordinary rain events,” which we defined as 1 day.

### Watershed-scale runoff modelling

To simulate runoff over the watershed, we coupled the KINematic Runoff and EROSion2 model (KINEROS2 or K2) and the Rangeland Hydrology and Erosion Model (RHEM) denoted as K2-RHEM. The K2 is a physically based watershed-scale hydrologic model developed by the Agricultural Research Service of the United States Department of Agriculture to simulate runoff in response to rainstorms at subdaily timescales (*56*, *57*). Modeled watersheds are represented by a network of overland-flow units, conceptualized as hillslopes, draining laterally into channels. Here, we use the latest version of the K2 that allows its integration with the hillslope-scale RHEM (*58*), a process-based model developed for undisturbed rangelands, where the impact of concentrated flow erosion is limited. The RHEM serves as a tool for runoff and erosion prediction at the hillslope scale, aiding in rangeland management and conservation assessment. Runoff routing in the K2-RHEM is based on the kinematic wave assumption and runoff generation takes into account various processes including varying rainfall intensity, rainfall interception by vegetation cover, surface storage, and infiltration (*58*–*60*).

### Model application and calibration

Watershed topographic data are based on the ALOS-PALSAR (*61*) DEM, with a 12.5-m resolution. We discretized the watershed into hillslope and channel elements using a contributing area threshold of 0.02 km^2^ based on a scaling break in the slope-area diagram of the watershed (*62*) and a qualitative comparison of the extracted channel network to the DEM and satellite images from Google Earth. To parameterize hillslope and channel elements, we used RHEM V2.4 parameter estimation equations (https://apps.tucson.ars.ag.gov/rhem/) that require topographic slope, vegetation/ground cover data, and soil texture information. We estimated vegetation and ground cover data based on aerial photos, satellite imagery, and field photos from Google Earth (fig. S13) for the six land-use types across the watershed based on the European Space Agency (ESA) land-use map (*63*). Soil texture was determined based on the iSDAsoil soil map (*64*). We set the initial soil moisture to 0.1 to represent a characteristic end-of-summer low value as there were no notable rain events over the watershed for more than 6 months before storm Daniel (fig. S8). The K2-RHEM model outputs were saved at a 10-min resolution.

As no hydrologic measurements are available for the Derna watershed, we calibrated the flood concentration time and volume based on the estimated runoff volume captured by the upstream dam. The dam height was determined by Ice, Cloud and land Elevation Satellite #2 (ICESat-2) Light Detection and Ranging (LiDAR) data (*65*), and the maximum fill volume was based on the ALOS-PALSAR DEM (fig. S1). Based on eyewitnesses, the dam filling started after 22:00 UTC, and the dam started to collapse sometime before 02:00 (*66*). Accordingly, we calibrated the K2-RHEM model to match the estimated volume at 23:30 ± 30 min using the relative bias as an objective function and by changing the hydraulic conductivity and roughness factors (the two main parameters governing the timing and the total discharge reaching the dam). We account for all hydrographs that show errors ≤ 5% of the best (minimal) objective function score as part of the uncertainty range. The results are reported as the median hydrograph and the minimum and maximal hydrographs of this uncertainty range (Fig. 2).

### Storm Daniel’s flood return period

To estimate the recurrence frequency of Storm Daniel’s flood, we compared the simulated flood volume to eight synthetic rainstorms and their resulting flood simulations. These rainstorms correspond to different daily rainfall return periods. We first computed the pixel-based daily rainfall for eight different return periods throughout the catchment (fig. S10, and the “Return period analysis” section in the Supplementary Materials). Then, for every return period, these pixel-based daily rainfall values were interpolated to half-hourly rain rates and used as input to the hydrological model to derive the corresponding flood volume. These synthetic storms represent an extreme case in which during a single storm, rainfall throughout the catchment is composed of the same return period; an uncommon situation given the high spatial variability of rainfall in this area, which may yield overestimated flood volumes. On the other hand, these synthetic storms are based on daily rainfall (interpolated to constant half-hourly rates) rather than subdaily intervals. Subdaily rainfall is typically associated with higher rainfall intensities, which, in turn, promote higher runoff coefficients, and thus, the flood simulations may result in underestimation. Overall, we suggest that our synthetic storms and the corresponding flood’s return level estimations are a good compromise, which is the best one could estimate given the uncertainty in this ungauged basin.

### Estimation of the dam’s spillway capacity

To estimate the potential discharge from the dam through the spillway we used Torricelli’s law. The law describes the velocity (*v*) a fluid obtains when flowing through an orifice with a specific hydraulic head (*H*). According to Torricelli’s law, the velocity is $v=\sqrt{2\mathrm{gH}}$, where *g* is the gravitational acceleration. The discharge from the same orifice can be calculated using its area (*A*) by *Q* = *Av* multiplied by 1 − *C*_d_, where *C*_d_ is the drag coefficient. We used two approaches to get the orifice diameter and the hydraulic head: (i) via estimates based on Google Earth imagery (fig. S15) and (ii) estimates previously published in (*4*). Both approaches yield discharge values smaller than 200 m^3^ s^−1^ (figs. S16 and S17).

### Flood scenarios modelling

We used the Hydrologic Engineering Center River Analysis System (HEC-RAS) two dimensional (2D) [version 6.4.1; (*67*)] to examine the flood consequences under two main scenarios: dam breach and no-dam. The optimal hydrograph from the K2-RHEM model was used as an input, 7 km upstream of the upstream dam. In addition, for each scenario, the K2-RHEM model hydrograph uncertainty range was simulated. The ALOS-PALSAR (*61*) DEM was locally edited to remove the two main dams in Wadi Derna. For the dam breach scenario, the upstream dam was re-inserted into the DEM using its measurements (fig. S1). Based on eyewitnesses’ descriptions of the breach (*66*), an overtopping failure was modeled. As the downstream dam was approximately 15 times smaller than the upstream one, we assume it was filled and collapsed rapidly. This assumption is reinforced by in situ measurements of flood watermarks 9 m above the downstream dam crest and the dam’s rapid and complete destruction (*68*). Therefore, the downstream dam was not simulated. If the downstream dam had been simulated in the no-dam scenario, then the resulting flood extent and SP in Derna would have been even smaller, leading to larger differences between the two scenarios.

To estimate Manning coefficients, we used the ESA land cover dataset (*63*). The coefficients were determined on the basis of the HEC-RAS 2D documentation v6.4 [(*67*) and table S3]. The main objective of the flood simulation was to compare the relative impact of the different scenarios rather than reconstructing their exact magnitude and extent, which is limited by the resolution of the available DEM. Therefore, our approach to evaluating the model results was based on the coarse extent of the floods compared to remotely sensed and in situ documentation of building destruction and flood depth [(*39*, *68*) and figs. S18, S20, and S21].

The HEC-RAS 2D SP is calculated as the average shear stress multiplied by the average flow velocity across each of the computational cell faces within the simulated area (*67*), i.e., it is equivalent to unit SP [W m^−2^]. Because the SP was calculated using water density of 1000 kg m^−3^, the resulting SP values are underestimations, as the water density of floodwaters is expected to be higher. Additional model specifications are described in the Supplementary Materials.

## Supplementary Materials

### The PDF file includes:

Supplementary Text Figs. S1 to S22 Tables S1 to S4 Legend for movie S1 References
