# Supplementary material for: Anatomy of a foreseeable disaster: Lessons from the 2023 dam-breaching flood in Derna, Libya
Source: Sci Adv. 2025 Mar 28;11(13):eadu2865. doi: 10.1126/sciadv.adu2865 (PMC11952095; doi:10.1126/sciadv.adu2865)
Supplement: Supplementary file 1 — Supplementary Text Figs. S1 to S22 Tables S1 to S4 Legend for movie S1 References [file sciadv.adu2865_sm.pdf]

Science Advances
AAAS

Supplementary Materials for

Anatomy of a foreseeable disaster: Lessons from the 2023 dam-breaching flood in Derna, Libya

Moshe Armon *et al.*

Corresponding author: Moshe Armon, moshe.armon@mail.huji.ac.il

Sci. Adv. **11**, eadu2865 (2025)
DOI: 10.1126/sciadv.adu2865

The PDF file includes:

Supplementary Text
Figs. S1 to S22
Tables S1 to S4
Legend for movie S1
References

Other Supplementary Material for this manuscript includes the following:

Movie S1

Supplementary Text

Dam volume estimation

The upstream Derna dam was built in the 1970s, meaning that sedimentation could change its designed maximum volume. To calculate the capacity of the upstream dam, we used the Advanced Land Observing Satellite instrument: Phased Array type L-band Synthetic Aperture Radar (ALOS-PALSAR; based on measurements from 12 June 2008) digital elevation model (DEM) at a 12.5 m/pixel resolution. This resolution is too coarse to capture the dam crest, which is <10 m wide. Therefore, to determine the dam height and the derived dam volume, we first corrected the DEM's crest height using the Ice, Cloud and land Elevation Satellite #2 (ICESat-2) satellite altimetry data from 8 July 2022 (Fig. S1). Combining the ICESat-2-derived crest height (254 meters above sea level; masl) with the ALOS-PALSAR DEM results in a dam capacity of 21.35 Million Cubic Meters (MCM). This volume is in line with the reported dam-designed capacity of 22.5 MCM (68). This small difference (5%) could be explained by sedimentation since the construction of the dam.

Meteorology

Surface wind speed

Wind speed over the Mediterranean Sea slightly before the maximum storm intensity (Fig. S2) was derived from satellite observations. We used the MetOp-C ASCAT Level 2 Ocean Surface Wind Vectors Optimized for Coastal Ocean product, which we obtained from the High-Level Tool for Interactive Data Extraction (HiTIDE V 4.10.0) at https://hitide.podaac.earthdatacloud.nasa.gov/.

Cyclone frequency

The frequency of cyclones in the vicinity of the study area (30°N – 34°N, 19°E – 23°E) was calculated based on binary cyclone mask fields (*33*). These cyclone fields are based on ERA5 sea level pressure maps from 1950–2023, in which cyclones are defined as enclosed regions containing one or more sea level pressure minima. The daily frequency from mid-summer to mid-winter is shown in Fig. S3, and the yearly monthly occurrence of cyclones is shown in Fig. S4.

Anomaly analyses

Anomalies of various meteorological parameters (Fig. S5) were derived from ERA5 data (see *Materials and Methods*).

Precipitation

Calibration

Satellite-based precipitation data was specifically calibrated to match the peak of the precipitation event in Cyrenaica. We calibrated Integrated Multi-satellitE Retrievals for the Global Precipitation Measurement mission (IMERG)-late V06 data (see *Materials and Methods*) to match daily precipitation published by the Libyan National Center for Meteorology on social media (in Arabic). These data are shown in table S1. Satellite values show a -38% bias compared to these gauge observations, as well as a 126 mm Root Mean Square Deviation (RMSD) error. To obtain a better fit between the satellite-based

precipitation and the ground-based observations, we used a two-step procedure (see *Materials and Methods*) in which we first shift the precipitation in space to obtain the best fit (18), and then remove the remaining bias. By examining a 5 × 5 box (±2 pixels at the zonal and meridional directions) we concluded that the minimum error in both bias and RMSD is obtained when shifting the precipitation field one pixel-step to the east and two to the south (Fig. S6), i.e., a $\sim 21\ km$ shift. We then eliminated the remaining mean field bias by dividing precipitation values by the (non-normalised) bias. The resulting accumulated precipitation during the peak of the event is shown in Fig. S7 and a time series of the mean areal precipitation from 06/2000 until the event is shown in Fig. S8.

Return period analysis

To estimate the return period of precipitation in the study area we used the daily resolution gauge-calibrated IMERG-final V06 data spanning 06/2000–05/2021. We used the Simplified Metastatistical Extreme Value (SMEV) method (35) on pixel-based data to derive the return periods. Intensity values for four locations across the Wadi Derna catchment based on SMEV are shown in Fig. S9, and regional maps of the intensity corresponding to different return periods are in Fig. S10. Daily precipitation during the peak of the event (Fig. S11a) is in the order of a few tens of years in terms of return periods (Fig. S11b). It reached nearly 200 mm at the northwestern side of the catchment, which translates into a return period of 77 years (Fig. S11b).

Hydrological model parameters

Elements of the coupled KINematic Runoff and EROSion2 model (KINEROS2 or K2) and the Rangeland Hydrology and Erosion Model (RHEM), denoted as K2-RHEM, were parameterised using the RHEM V2.4 parameter estimation equations (https://apps.tucson.ars.ag.gov/rhem/docs) that require topographic slope, soil texture, and vegetation and ground cover data. The topographic slope for every model element has been determined based on the average slope among all of the pixels within each element. The soil texture per element was determined by the texture category exhibited in the majority of pixels within the element (Fig. S12). To estimate vegetation and ground cover parameters, we first estimated a characteristic vegetation and ground cover for each land use category based on satellite imagery from Google Earth (Fig. S13 and table S2). Then, we calculated
the vegetation and ground cover parameter per model element according to the relative proportion (number of pixels) of all land use categories within the element (see Fig. S12).

Estimating the dam's spillway capacity

To estimate the output discharge from the dam's bell-mouth spillway we used estimates for Torricelli's law parameters (see *Materials and Methods*) from two sources: (a) Google Earth imagery (Fig. S15), and (b) ref. (4). We estimate the hydraulic head of the water to be <5 m, while ref. (4) concludes it is 2.5 m. We show discharge estimates using different drag coefficient values based on these two different parameter sets (Fig. S16). Additionally, we show the expected discharge based on different combinations of hydraulic heads and orifice diameters (Fig. S17) using a conservative drag coefficient ($C_d = 0.05$). Based on this analysis we can conclude that the potential discharge by the spillway is $< 200\ m^3\ s^{-1}$.

Hydraulic flood simulations

We used Hydrologic Engineering Center River Analysis System (HEC-RAS) 2D simulations to evaluate the downstream impact of the storm-generated flood, based on the output of the K2-RHEM hydrological model. Given the limitations of the DEM resolution (12.5 m/pixel) and the uncertainty regarding the timing and mechanism of the dam breaches, the goal of the hydraulic modelling was to compare the difference between the scenarios, rather than precisely reconstruct the flood extent and magnitude. This use of 2D HEC-RAS to constrain flood impacts is in line with previous modelling of extreme floods (70-73). Two main scenarios were simulated: dam breach and no-dam in Wadi Derna. We also modelled scenarios of dam overflow without breaching and a longer dam breach (table S4, and movie S1). While the simulated peak discharge of the no-breach scenario is the smallest of all scenarios ($649\ m^3\ s^{-1}$), the flood area and depth in Derna in this scenario are similar to those of the no-dam scenario. The longer dam breach simulation (1.5 hours) yielded flood extent and maximum depth similar to the outcomes of the shorter breach scenario (0.5 hour), but exhibited a 20% decrease in peak discharge.

We used a cell size of 20 m for the computational mesh downstream of the upstream dam and 50 m per cell for the upstream area. Manning coefficients were set to vary with land use (table S3) according to values suggested by ref. (67). For stable and robust model runs, we used the Diffusion Wave Equations, and computation steps of 1 second. The two Derna dams were manually removed from the DEM. The upstream dam was re-digitized in the breach and overtopping (no-breach) scenarios. In contrast, the downstream dam was assumed to be insignificant for comparing scenarios and under the simulated flood discharge and volumes, as evident in situ by documentation of flood watermarks 9 meters above the dam, and its rapid and complete destruction (68). The upstream dam measurements before and after the breach were based on aerial imagery (MAXAR), Light Detection and Ranging (LiDAR) data (ICESat-2; Fig. S1), and field measurements and images based on ref. (68). The duration of the breach progression was estimated using the HEC-RAS breach parameter calculator, yielding a range of 0.6–1.5 hours. Based on the reported poor mechanical conditions of the dam, we used 0.5 hours as the main breach duration. This duration aligns with the timing reported by eye-witnesses (66). For validation, model results were compared to in situ flood watermarks on buildings in the City of Derna from ref. (68). As noted, we did not intend to reconstruct precise flood depths due to the DEM resolution. However, the simulated depths show a positive correlation with these watermarks (fig. S18).

The HEC-RAS 2D stream power (SP) is calculated as the average shear stress multiplied by the average flow velocity across each of the computational cell faces within the simulated area (67). I.e., it is equivalent to unit stream power [$W\ m^{-2}$]. For verification, we multiplied the shear stress output raster [$Pascal$] by the velocity output raster [$m\ s^{-1}$], resulting in similar values to the HEC-RAS 2D SP output. In all figures and tables in the main text and supplementary material, *stream power* refers to *unit stream power*. A comparison between the depth and SP of the simulated breach flood to the damaged buildings in Derna (41) pointed to a SP value of $1000\ W\ m^{-2}$ as a threshold for significant damage (fig. S20 and Fig. 4 in the main text). This threshold further emphasises the significant differences between the consequences of the two flood scenarios. While the destructive flood (SP values greater than the threshold) hit extensive neighbourhoods in Derna during the flood, in the no-dam scenario, these high SP values were restricted to the canal crossing the city, with very few buildings in it (fig. S21).

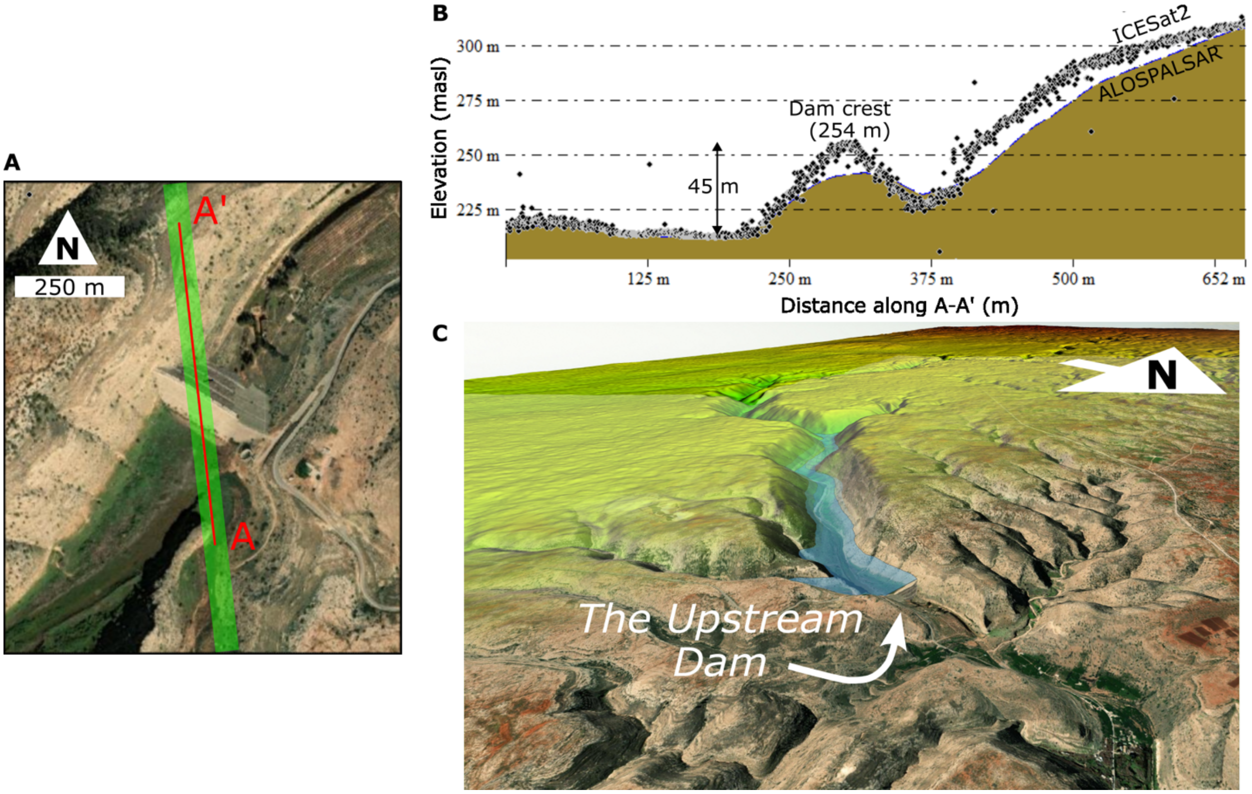

Fig. S1. Estimation of the upstream dam capacity.

Maximum volume calculations are based on the ALOS-PALSAR DEM and ICESat-2 LiDAR data points. (A) The location of the ICESat-2 altimeter beam (8 July 2022) and the corresponding A-A' cross-section along the upstream dam. (B) The dam height differences are compared between the ALOS-PALSAR DEM and ICESat-2 elevation data along the A-A' cross-section. (C) The resulting maximum dam storage extent, 21.35 MCM, on top of the ALOS-PALSAR DEM and MAXAR imagery (9 May 2022, 1.2 m/pixel).

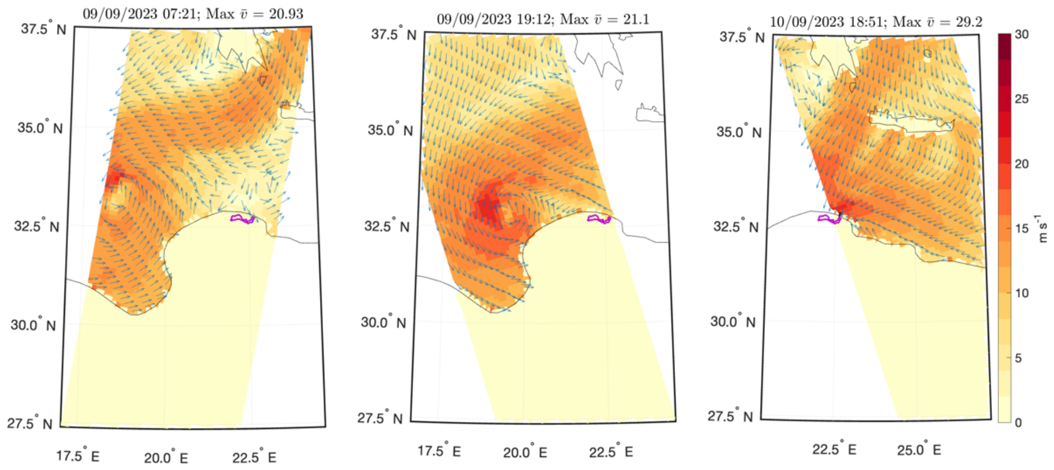

Fig. S2. Wind over the Mediterranean Sea slightly before the storm's peak intensity. Colours (over the ocean) show wind speed and arrows show its direction. Panel titles describe the timing of every snapshot and the maximum observed wind speed throughout the region shown.

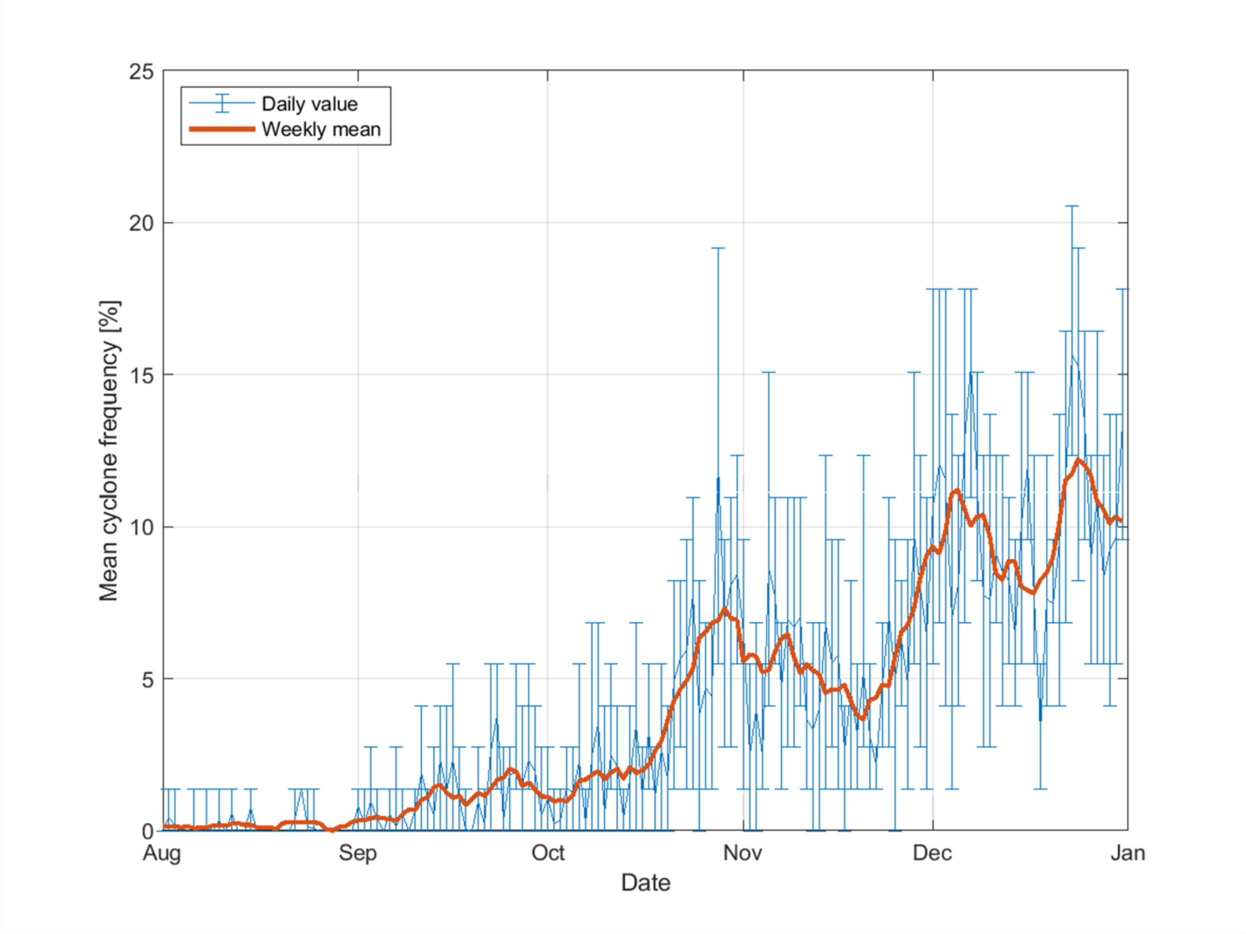

Fig. S3. Frequency of cyclones in the vicinity of the study area, 1950–2023.

Mean daily values are shown in a blue line, and its 7-day average is in bold orange line. Error bars represent minimum and maximum values across the region.

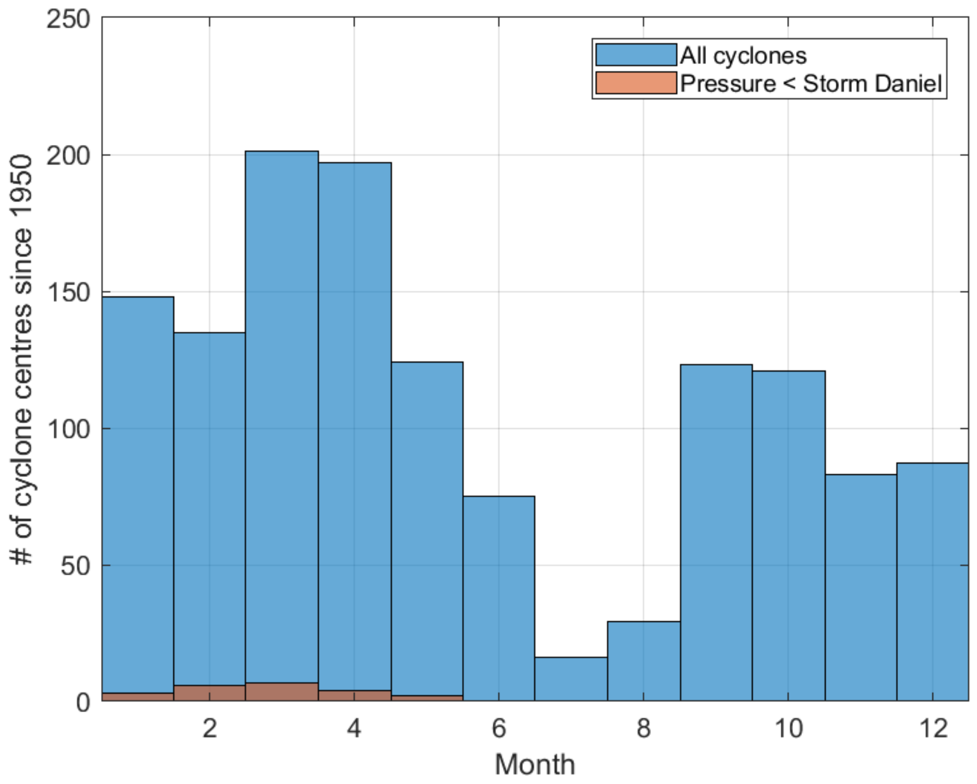

Fig. S4. Monthly occurrence of cyclones in the vicinity of the study area.
Cyclones with minimum pressure lower than the pressure during Storm Daniel are marked in orange.

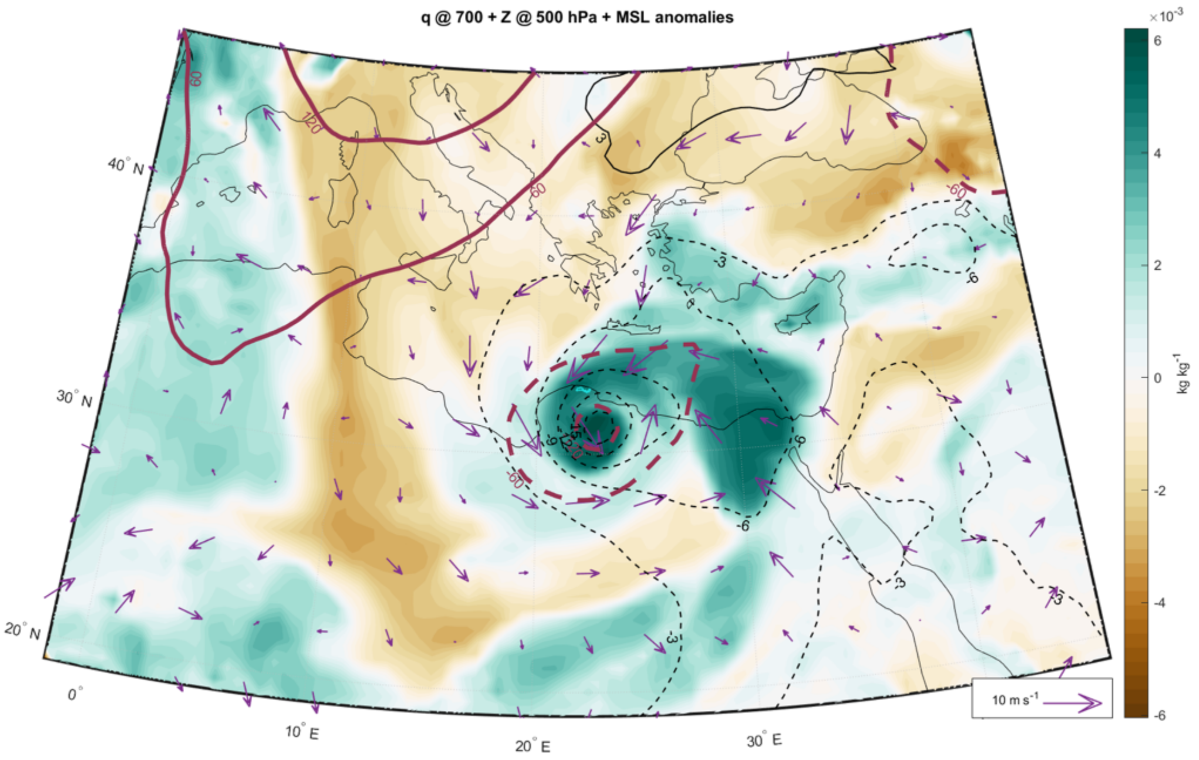

Fig. S5. Atmospheric anomalies during the most intense phase of Storm Daniel (11 September 2023, at 00:00 Coordinated Universal Time; UTC).

Anomalies are computed versus mean September values (at midnight). Specific humidity at 700 hPa is coloured, mean sea level (MSL) pressure is shown with black contours (dashed contours denote negative values), and 500 hPa geopotential height is shown with thick red contours (dashed where negative). Near-surface (10 m) wind anomalies are shown with purple arrows. The catchment of Wadi Derna is in Cyan.

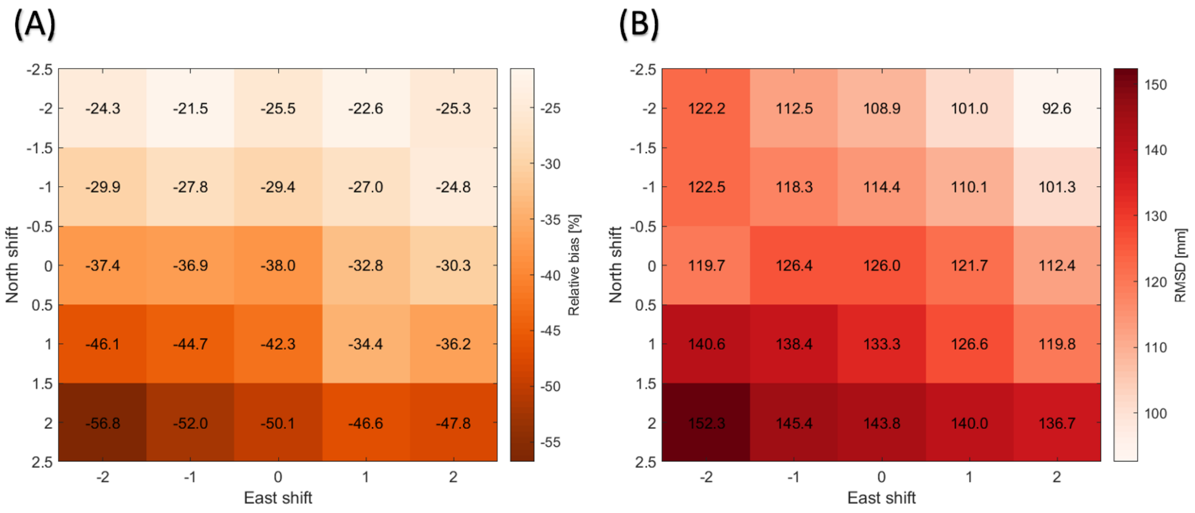

Fig. S6. Error values when shifting the IMERG precipitation field in space.

Relative bias (a), and RMSD (b) matrices are shown with respect to the eastward (x-axis) and northward (y-axis) shift of the precipitation field. To minimize the error, we shifted precipitation one-pixel step eastward and two-pixel steps southward.

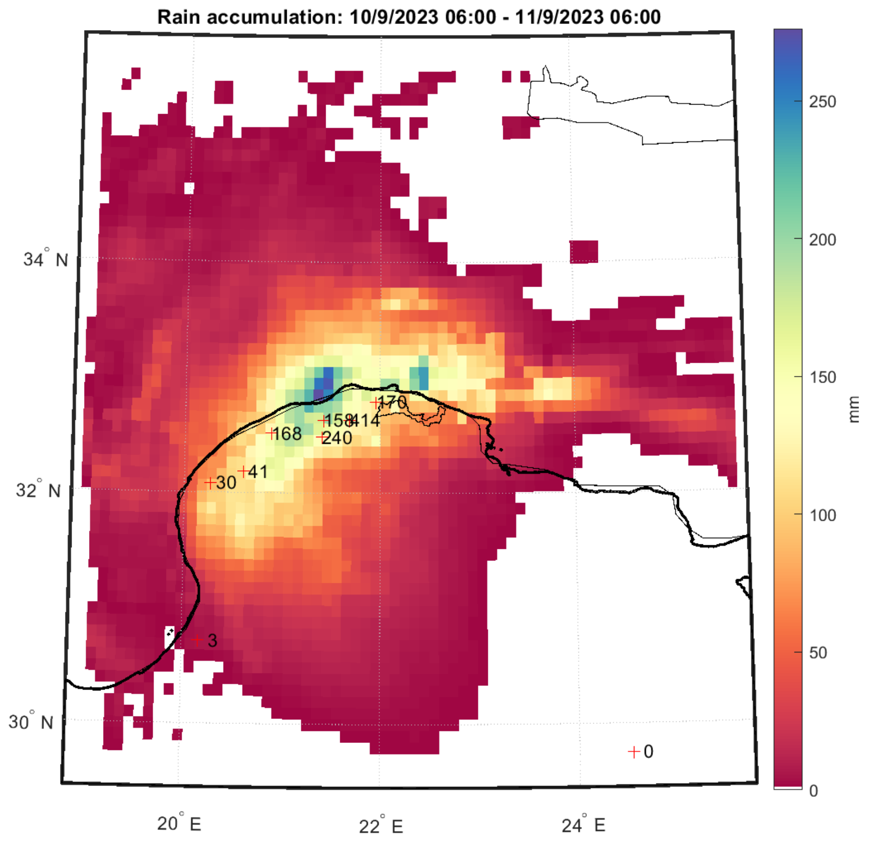

Fig. S7. Accumulated precipitation during the peak of the event (24 h ending on 11 Sep 2023, 6:00 UTC).

Colours represent IMERG post-calibration precipitation. Red crosses are gauge-based precipitation values (table S1). The Wadi Derna catchment is outlined in black.

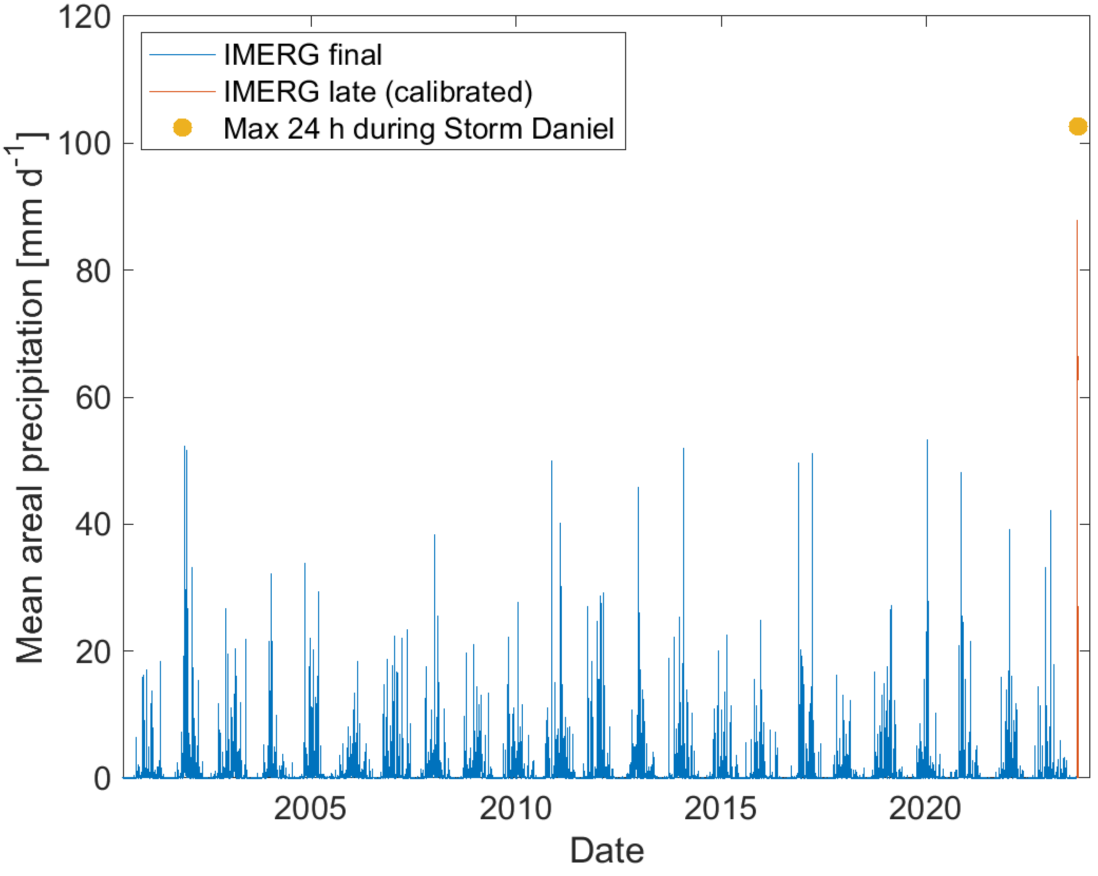

Fig. S8. Daily mean areal precipitation in the vicinity of the Wadi Derna catchment (within a box area with corners at 32.0829°N–33.3127°N, 21.4552 °E–23.1739 °E).

Values in blue represent daily (00:00–23:59 UTC) precipitation from IMERG-final data. The orange bars represent daily precipitation during Storm Daniel after calibration (see *Materials and Methods*, and the *Calibration* section above). The orange dot is the maximum 24 h precipitation, based on the half-hourly calibrated IMERG-late data ending on 11 Sep 2023, 6:00 UTC.

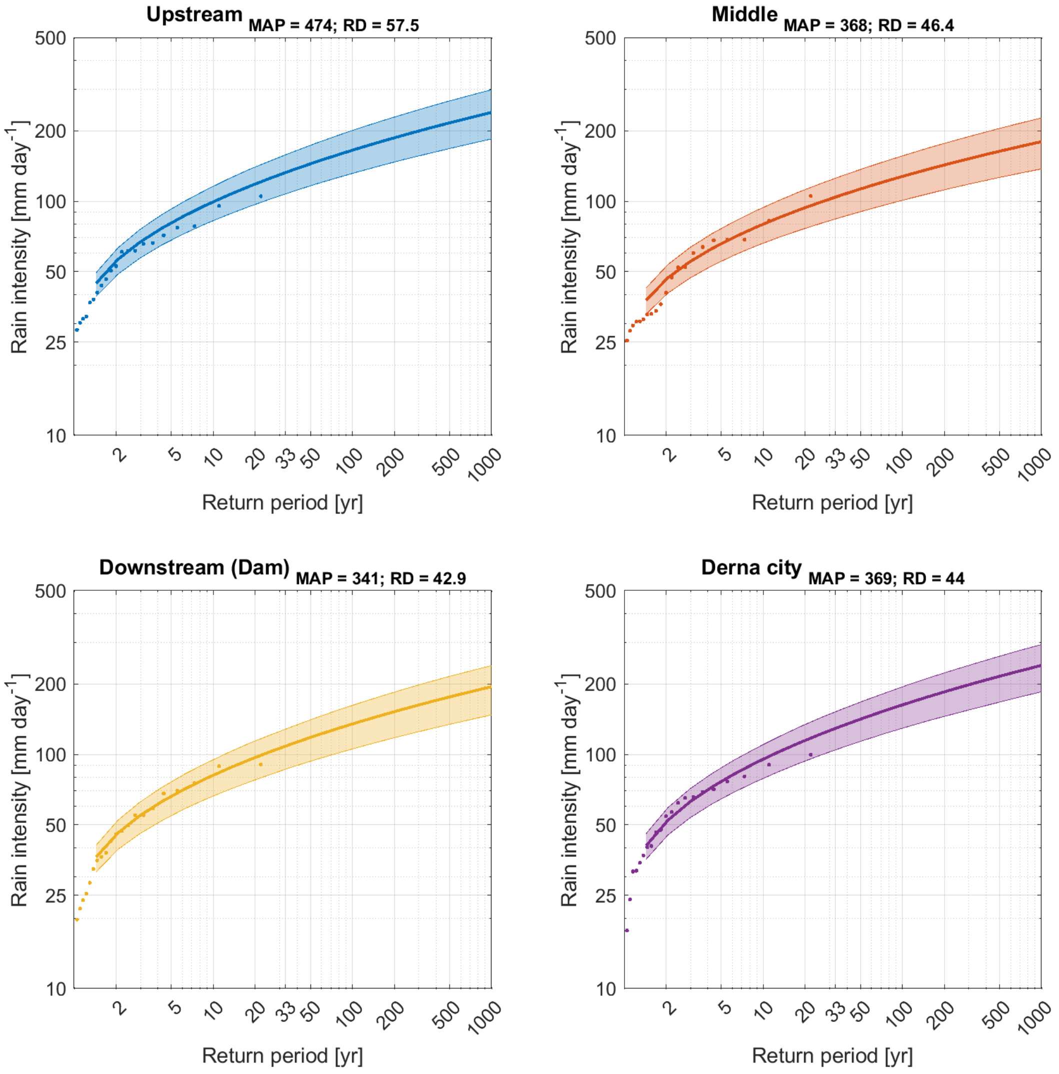

Fig. S9. Return period analysis for four locations across the Wadi Derna catchment. Dots represent empirical (satellite-based) data, while the curve is the SMEV estimate of the daily rain intensity for different return periods. Panel titles show the location, mean annual precipitation (MAP), and the number of rain days per year (RD) at every location. The locations are (32.7344°N, 22.0543°E), (32.6772°N, 22.3166°E), (32.6592°N, 22.5773°E), (32.7623°N, 22.6417°E), for the upstream, middle, downstream, and Derna city parts of the catchment, respectively.

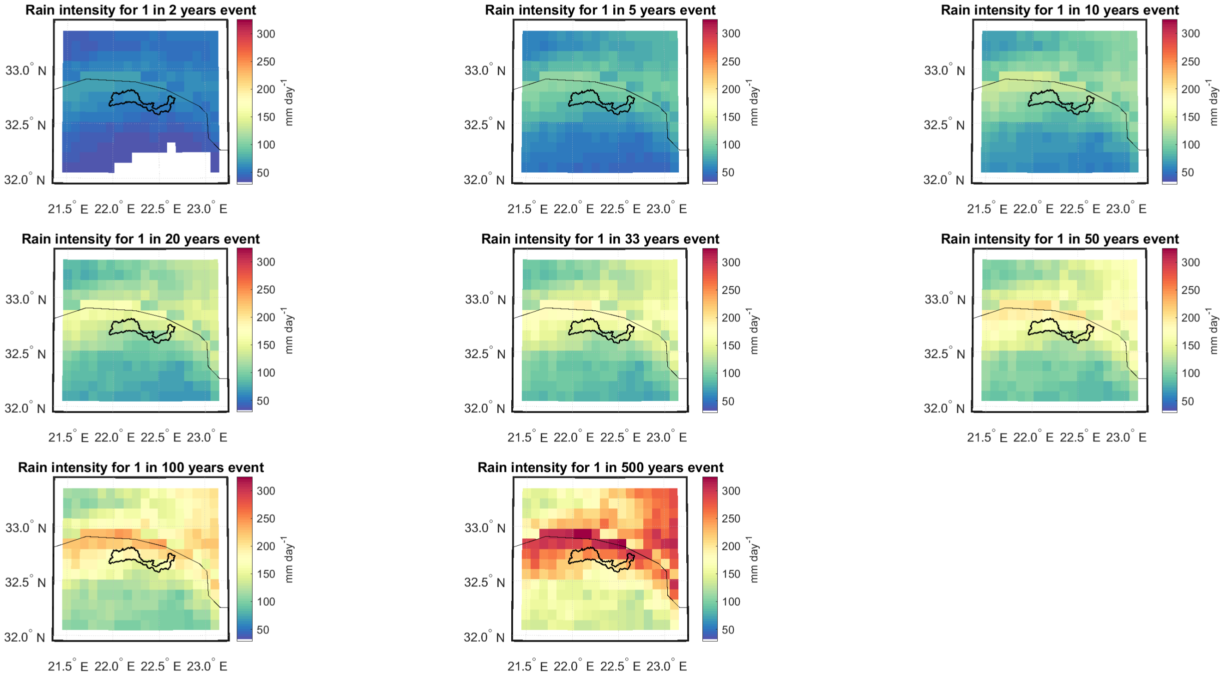

Fig. S10. Daily precipitation return period analysis for the Wadi Derna catchment region. Panel titles show the return period, and the catchment is outlined in black.

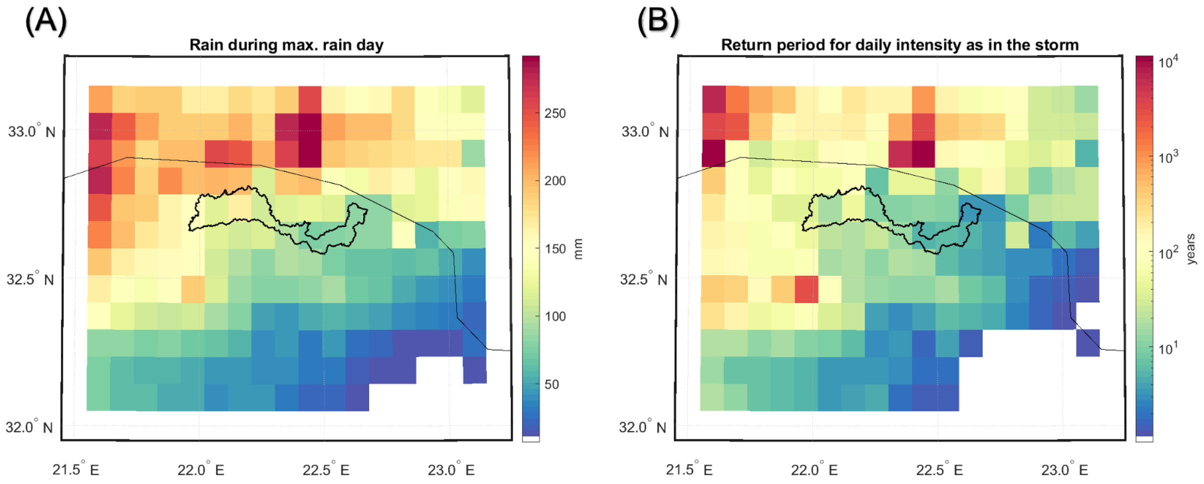

Fig. S11. Rainfall extremeness during Storm Daniel.

Daily calibrated rainfall during the peak of the event (a), and its translation into return periods (b). The Wadi Derna catchment is outlined in black. The highest pixel value exhibited over the catchment (in its northwestern side) is 77 years.

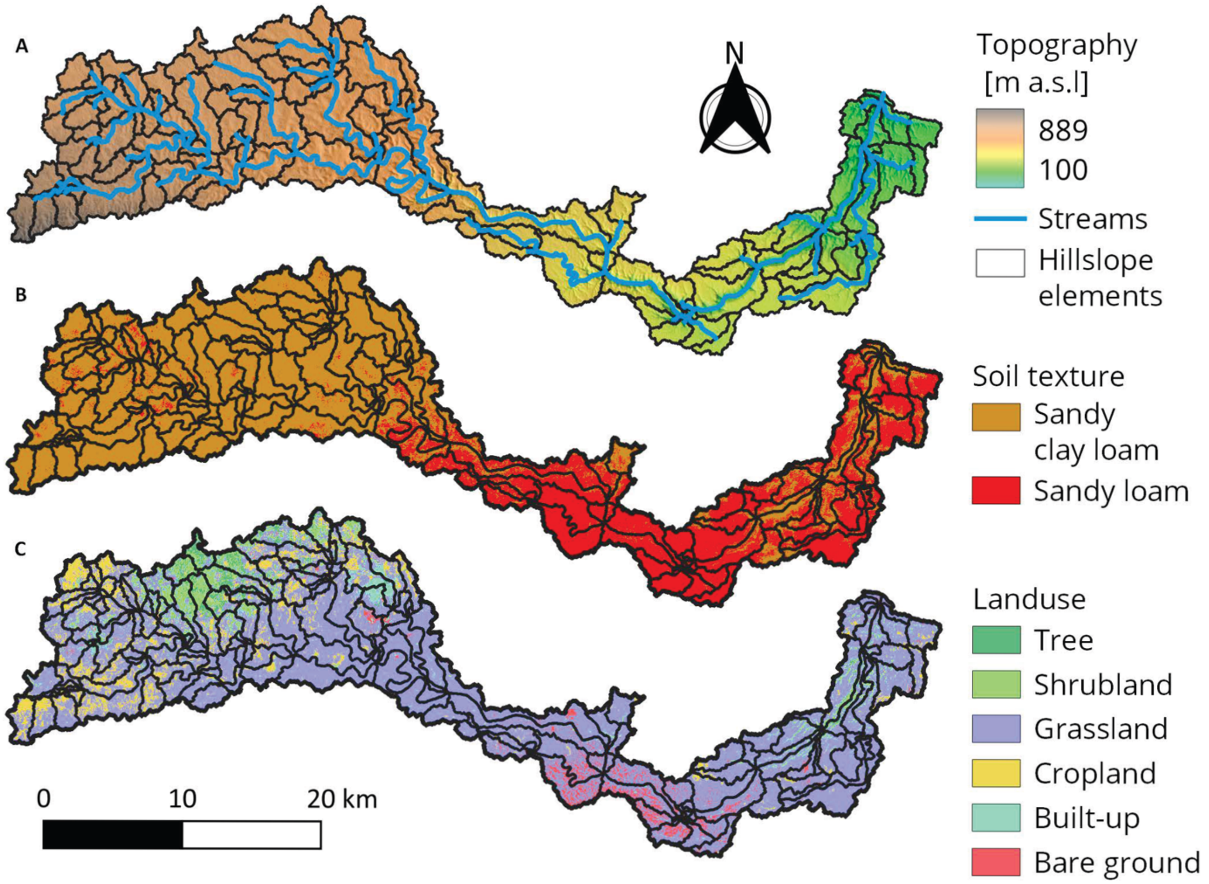

Fig. S12. Derna watershed properties.

(A) Topographic map. (B) Soil texture map. (C) Land use map. Black polygons are the hillslope model elements.

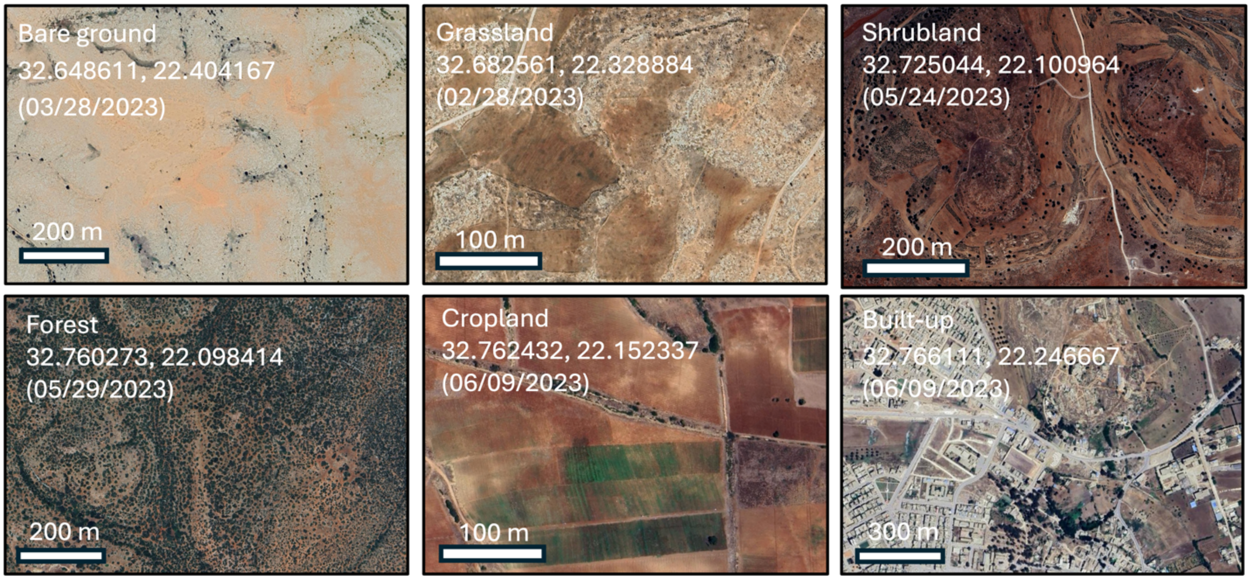

Fig. S13. Satellite images presenting various land use categories within the Derna watershed.

For each land use category, vegetation and ground cover parameters were evaluated based on satellite images from Google Earth (see table S2). Image data: Google, Airbus.

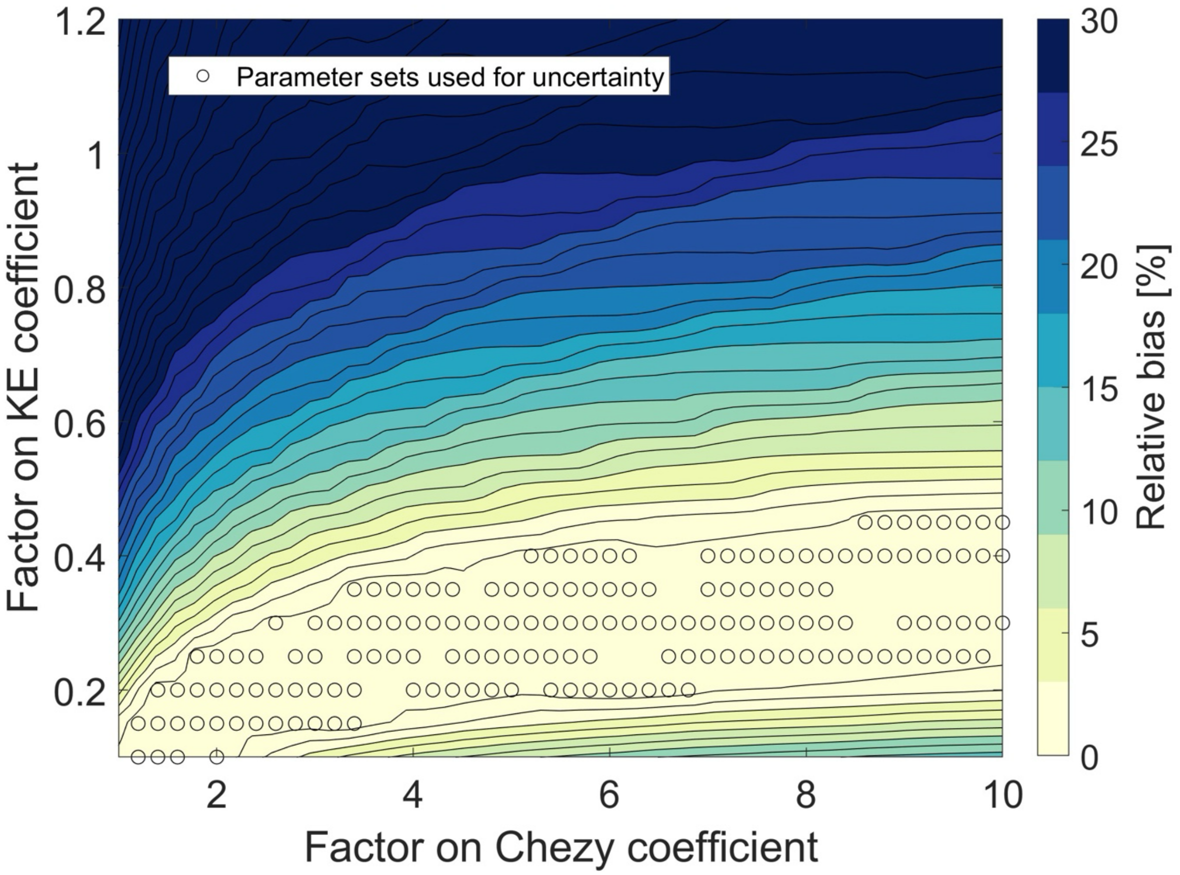

Fig. S14. Hydrologic model calibration.

A target surface describing changes in the value of the objective function (relative bias) with changes in factors on hillslope elements' Chezy and hydraulic conductivity (KE) coefficients. The relative bias is calculated as follows: $\frac{Q_s - Q_o}{Q_o} \cdot 100$, where Q_o is the estimated volume captured by the dam and Q_s represents the simulated total discharge volume within 30 minutes of the estimated breach time (30 minutes before and after) at the dam location. Within this 60-minute window, we chose the Q_s with the closest value to Q_o.

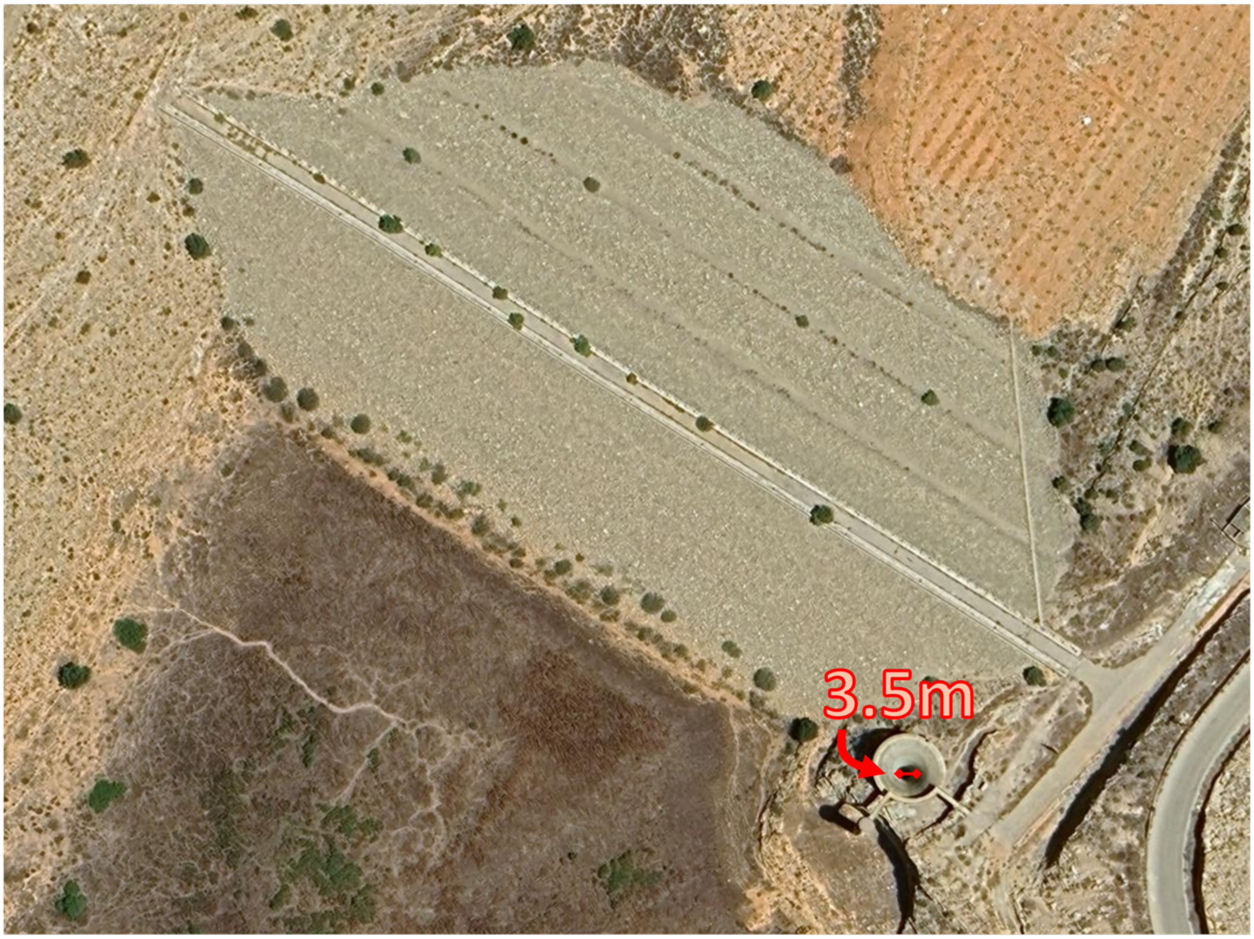

Fig. S15. Spillway diameter estimate from Google Earth imagery.
Image (Google, Airbus) taken on 19 June 2023, at 32.6583°N, 20.5769°E.

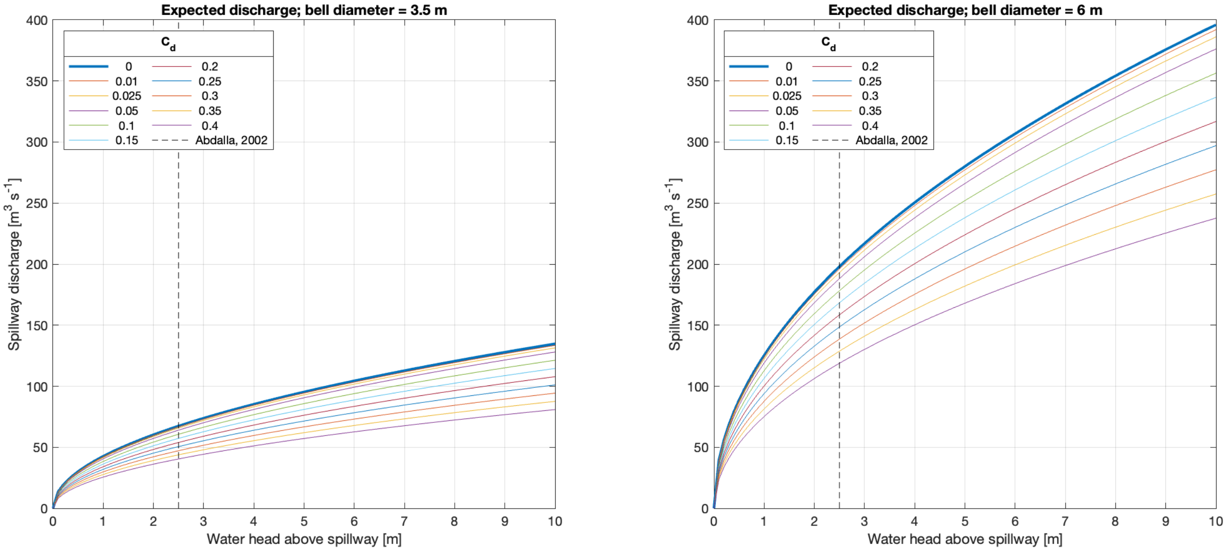

Fig. S16. Expected spillway discharge.

Discharge calculated by Torricelli's law for different drag coefficients (see *Materials and Methods*) is shown versus the hydraulic water head for an orifice diameter as estimated by Google Earth imagery (Fig. S15; left panel), and as concluded in ref. (*4*; right panel), where the water head is stated to be 2.5 m.

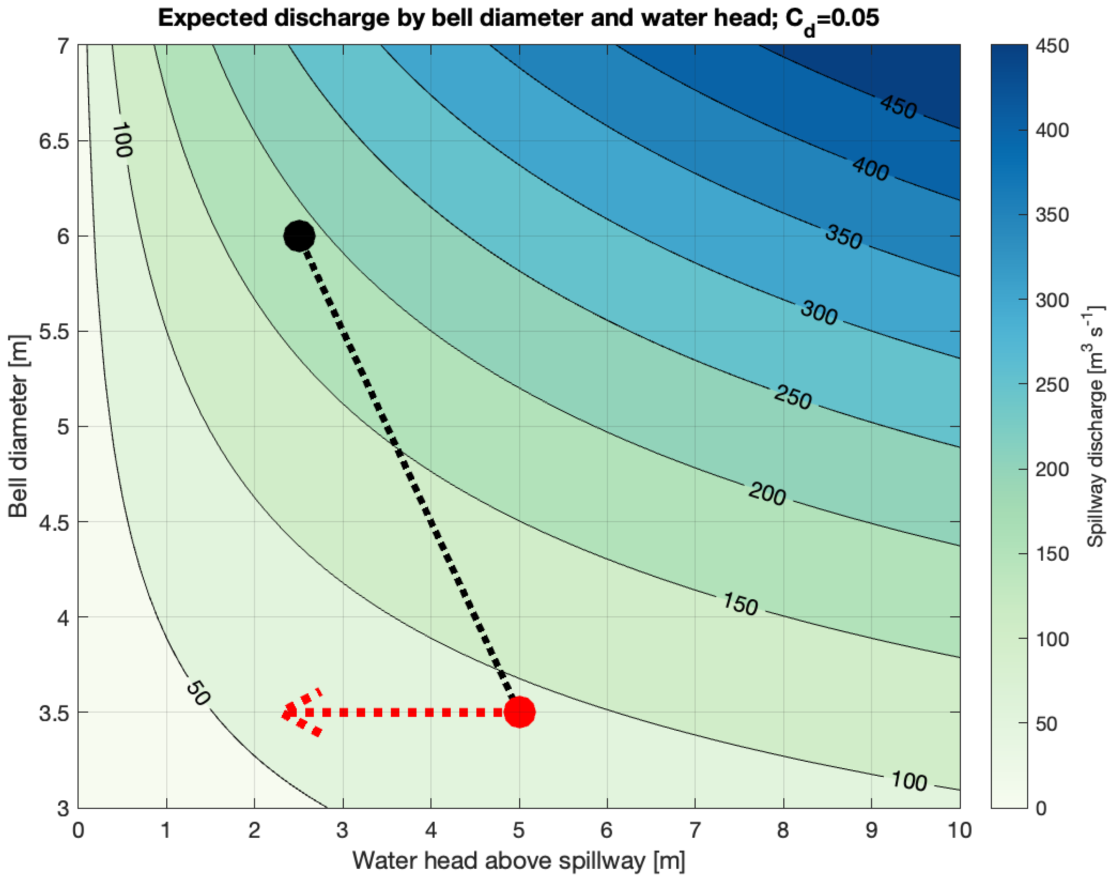

Fig. S17. Estimates of the spillway discharge.

Discharge calculated by Torricelli's law using a conservative drag coefficient ($C_d = 0.05$) is shown for different orifice diameters and hydraulic water heads (see *Materials and Methods*). Our estimates, based on Google Earth imagery are water heads of <5 m and an orifice diameter of 3.5 m (red symbol). Estimates given in ref. (4) are shown with a black dot. The actual discharge lies within the black-red triangle or lower, in case C_d is higher.

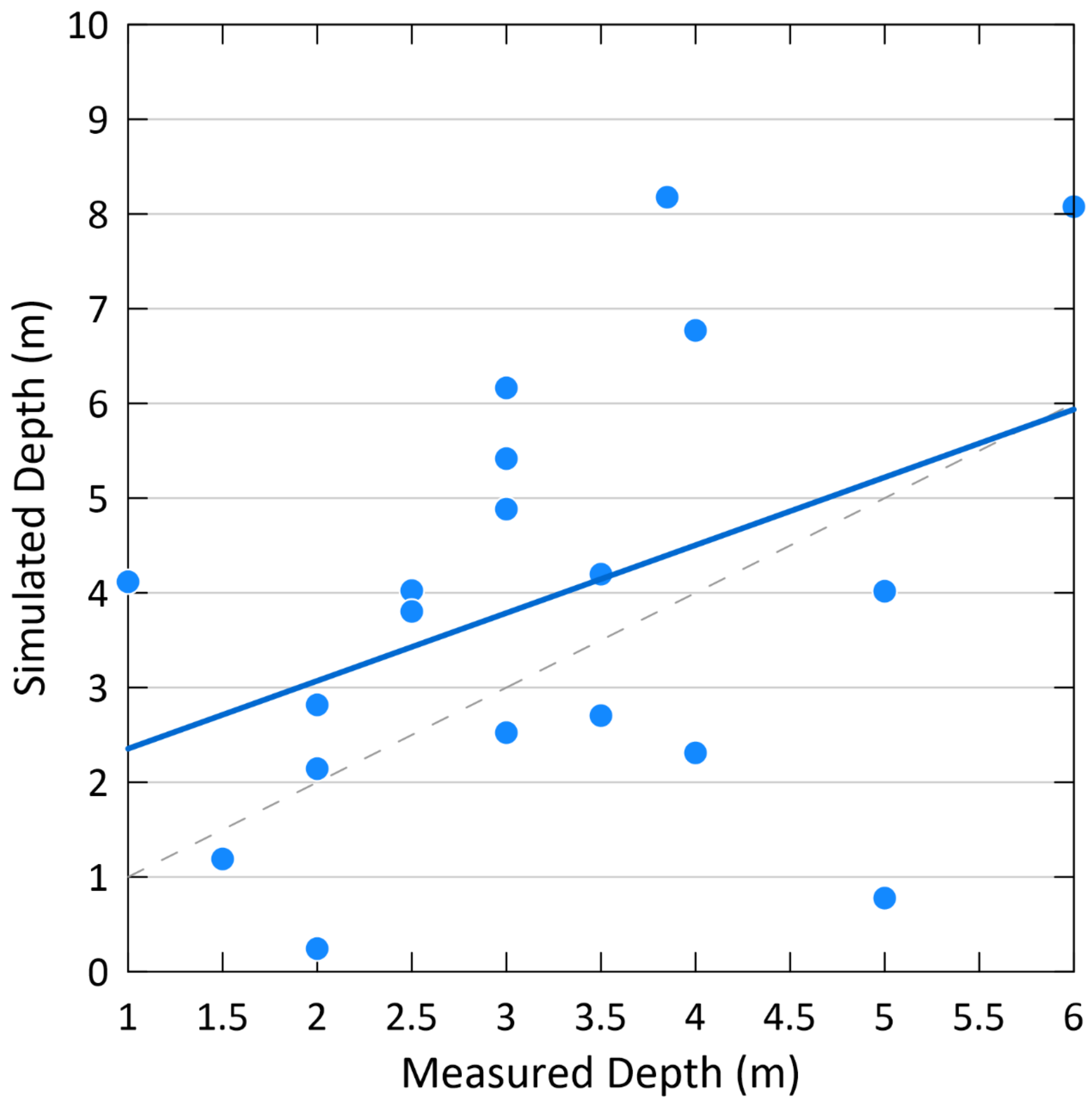

Fig. S18. Simulated breach flood depth vs. measured flood depths on buildings in Derna according to ref. (68)**.**

The dashed line is the 1:1 line, and the blue line is a linear regression ($R^2 = 0.87$). One data point, representing an extreme outlier in the observed data (12 m depth) was removed from this analysis.

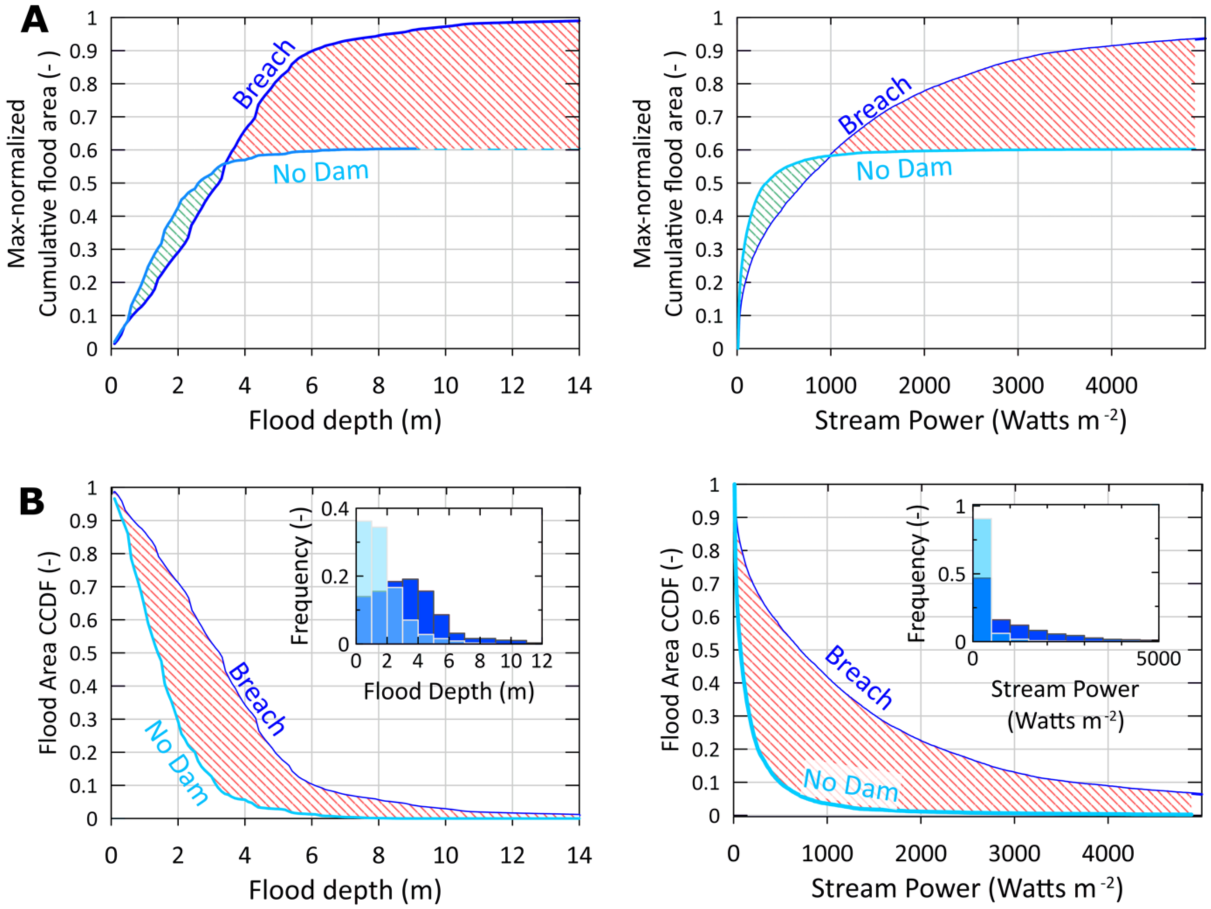

Fig. S19. Area distributions of the simulated maximum depth and (unit) stream power (SP) during the Derna flood under the scenarios of a dam breach and no dams.

(A) Cumulative flood area normalized to the maximum area in the breach scenario. (B) Complementary cumulative distribution function of the two scenarios.

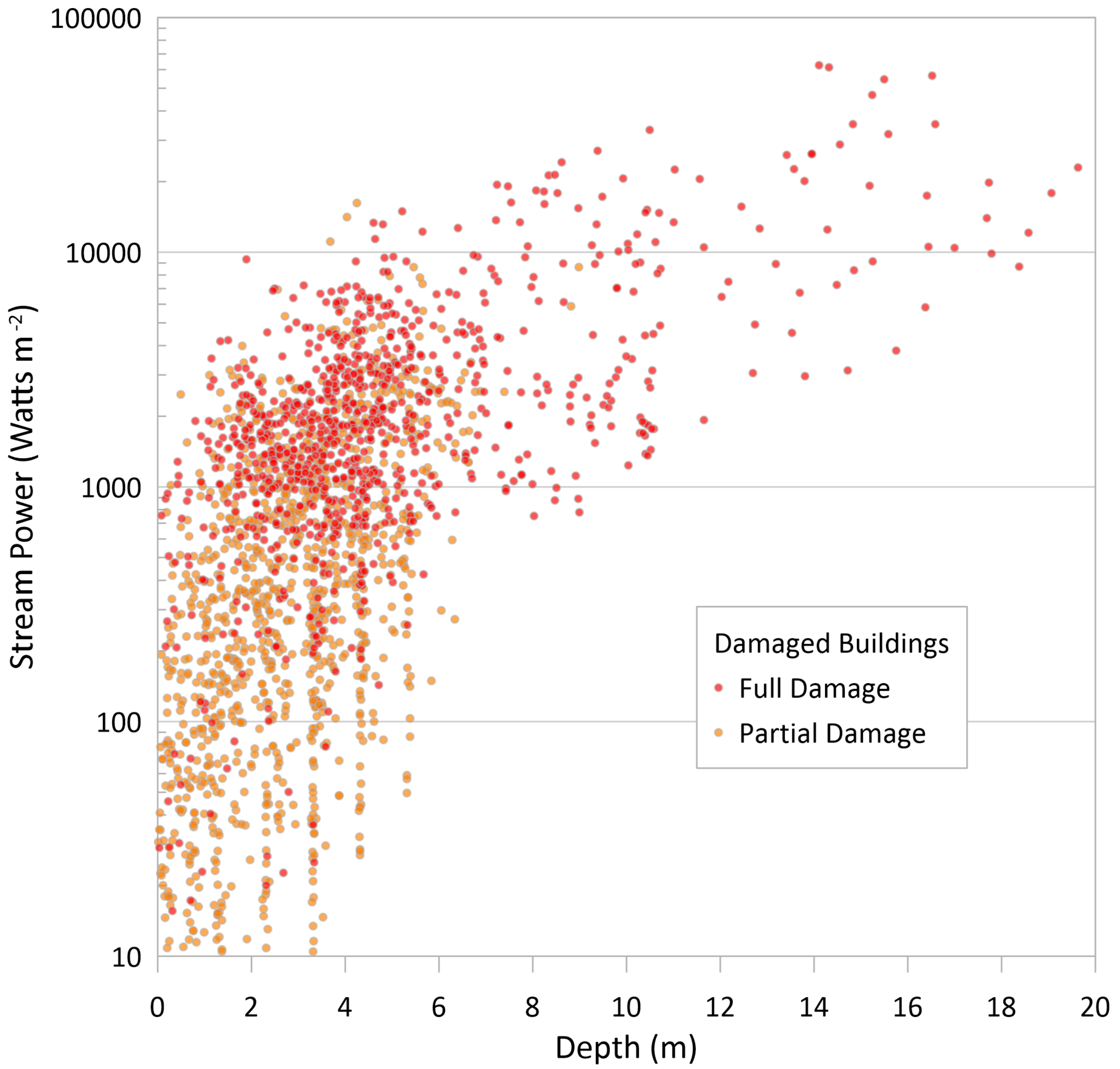

Fig. S20. Stream power and depth of the simulated dam breach flood.

Data were sampled where damage to buildings was reported. The building damage classification, determined by ref. (41), points to a (unit) stream power threshold of 1000 $W\ m^{-2}$ for significant infrastructure damage.

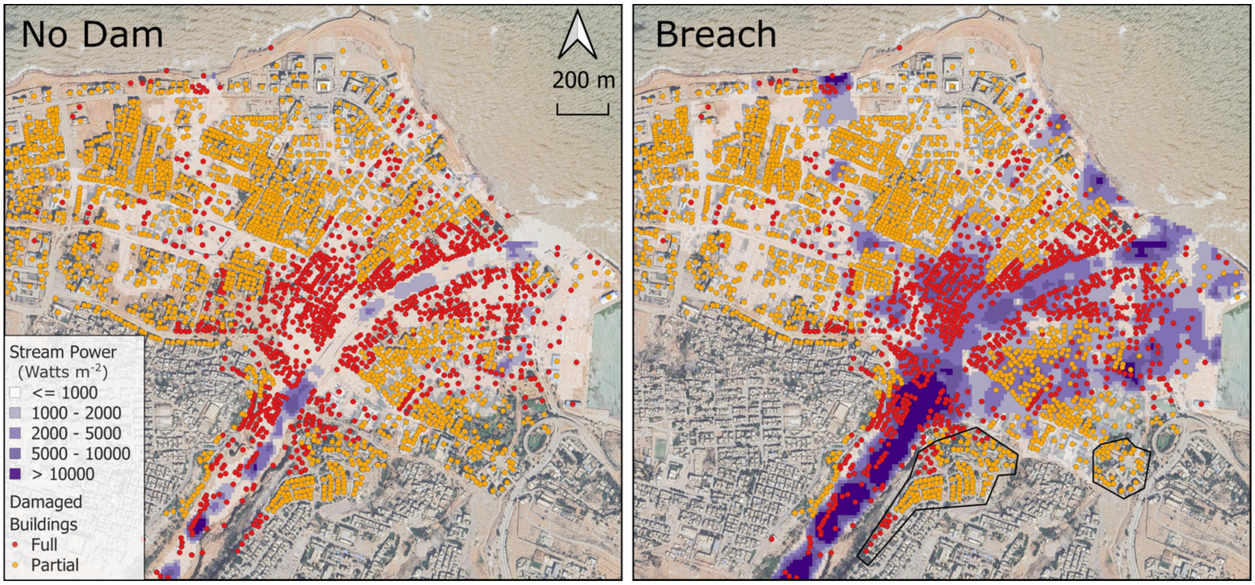

Fig. S21. Simulated flood maximum (unit) stream power for the no-dam and breach scenarios.

While in the breach scenario SP values $> 1000\ W\ m^{-2}$ prevail in the extent of the city and are co-located with buildings that were fully damaged by the actual flood, the no-dam scenario exhibits such values mainly within the extent of the Derna canal, with very few populated buildings. Damaged buildings that were probably affected by flooding from adjacent small streams (not modelled in the simulation) are marked within a black outline in the Breach results map. Background imagery: Google, Maxar Technologies (13 September, 2023).

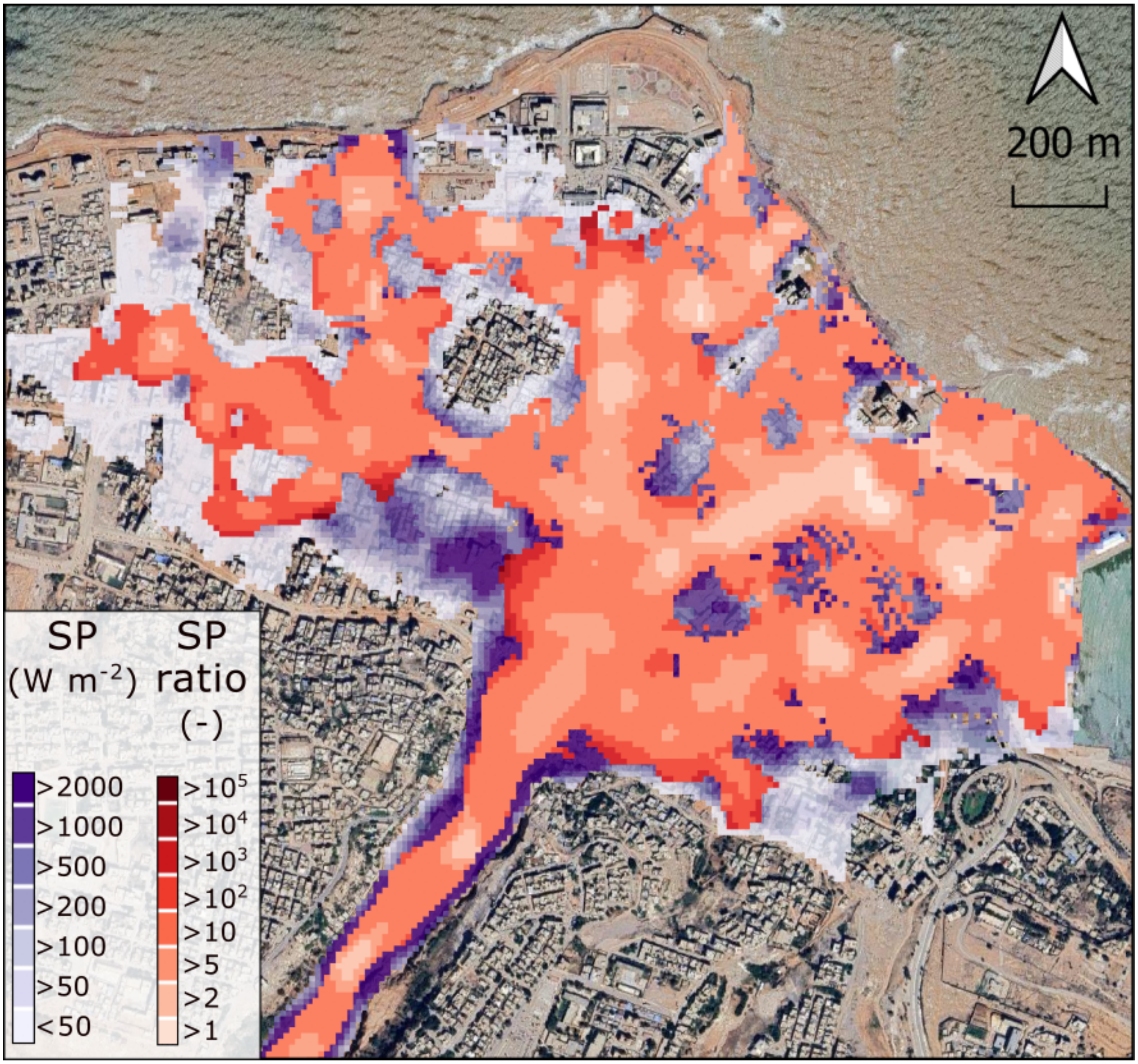

Fig. S22. Stream power (SP) ratios between the two flood scenarios (breach and no-dams). The SP of the breach scenario is in purple, whereas the red scale denotes the SP ratio (breach/no-dam) where the flooded area of the scenarios overlaps. Background imagery: Google, Maxar Technologies (13 September, 2023).

Table S1. Accumulated precipitation for the 24 *h* ending on 11 Sep 2023, 6:00 UTC. Data published on social media by the Libyan National Center for Meteorology.

Station	Latitude [°N]	Longitude [°E]	Precipitation [mm]
Al-Abyar	32.183	20.600	41
Al-Marj	32.517	20.883	168
Omar Al-Mukhtar (Al-Bayda University)	32.633	21.683	414.1
Al-Jaghbub	29.750	24.533	0.3
Ajdabiya	30.717	20.167	3.2
Benina airport	32.083	20.267	30
Qasr Libya	32.633	21.417	158.5
Al-Abraq airport	32.792	21.956	170
Marawa	32.483	21.400	240

Table S2. Vegetation and ground cover data for land use types in the Derna watershed. The vegetation and ground cover properties used to parametrize the K2-RHEM hydrological model are based on the RHEM parameter estimation equations v2.4.

Land use	Litter [%]	Soil crust [%]	Basal [%]	Rock [%]	Total ground cover [%]	Annual and forbs [%]	Bunch grasses [%]	Shrubs [%]	Sod grasses [%]	Total foliar cover [%]
Shrubland	10	10	10	60	90	10	10	10	0	30
Grassland	10	10	10	40	70	5	5	5	0	15
Forest	10	5	5	50	70	10	10	20	0	40
Bare	5	5	5	50	65	2	2	2	0	6
Built-up	10	0	5	80	95	10	10	10	0	30
Cropland	20	5	10	40	75	15	0	0	15	30

Table S3. Manning's coefficients used in the HEC-RAS 2D simulations. The values are based on the mapping by ref. (63) (see Fig. S12) and corresponding manning coefficients suggested by ref. (67).

Land Use	Manning's Coefficient
Grassland	0.04
Cropland	0.05
Bare	0.03
Built-up	0.15
Tree Cover	0.07
Shrubland	0.05

Table S4. Results of the flood scenarios, simulated in HEC-RAS 2D. Peak discharge was sampled near the downstream dam, and the maximum flooded area and depth were calculated within the city of Derna.

Scenario	Peak discharge (m^3 s^{-1})	Max. Flooded area (10^3 m^2)	Max. Flood depth (m)
0.5 hr. Breach	12,413	2,682	16.4
1.5 hr. Breach	9,810	2,598	15.1
No Breach	649	1,509	6.6
No Dam	1,386	1,838	6.7

Movie S1. Derna flood HEC-RAS 2D simulations. Colours denote flood depth (0-15 meters). Hydrographs were sampled near the Upstream Dam and the City of Derna.

REFERENCES AND NOTES

1. S. N. Jonkman, Global perspectives on loss of human life caused by floods. *Nat. Hazards* **34**, 151–175 (2005).

2. N. J. Middleton, T. Sternberg, Climate hazards in drylands: A review. *Earth Sci. Rev.* **126**, 48–57 (2013).

3. M. Evenari, D. Koller, Ancient masters of the desert. *Sci. Am.* **194**, 39–45 (1956).

4. M. G. Abdalla, Mathematical model of dam Break: Applied study of derna dam. *Mansoura Eng. J.* **27**, 14 (2002).

5. A. A. R. Ashoor, Estimation of the surface runoff depth of Wadi Derna Basin by integrating the geographic information systems and Soil Conservation Service (SCS-CN) model. *J. Pure Appl. Sci.* **21**, 90–100 (2022).

6. D. Sharon, The spottiness of rainfall in a desert area. *J. Hydrol.* **17**, 161–175 (1972).

7. D. Shohami, U. Dayan, E. Morin, Warming and drying of the eastern Mediterranean: Additional evidence from trend analysis. *J. Geophys. Res. Atmos.* **116**, D22101 (2011).

8. S. E. Nicholson, *Dryland Climatology* (Cambridge Univ. Press, 2011).

9. A. D. Knighton, G. C. Nanson, An event-based approach to the hydrology of arid zone rivers in the Channel Country of Australia. *J. Hydrol.* **254**, 102–123 (2001).

10. Y. Farhan, A. Anbar, Fragile Landscape: Impact and consequences of May 2014 flash-flood disaster in the Aqaba area, Southern Jordan. *Res. J. Environ. Earth Sci.* **6**, 451–465 (2014).

11. J. A. Smith, A. A. Cox, M. L. Baeck, L. Yang, P. Bates, Strange floods: The upper tail of flood peaks in the United States. *Water Resour. Res.* **54**, 6510–6542 (2018).

12. OCHA, Libya: Flood Response Humanitarian Update (as of 20 March 2024) (2024).

13. D. Delforge, V. Wathelet, R. Below, C. L. Sofia, M. Tonnelier, J. A. F. Loenhout, N. Speybroeck, EM-DAT: the Emergency Events Database. [Preprint (Version 2)] (13 January 2025). available at Research Square https://doi.org/10.21203/rs.3.rs-3807553/v2.

14. T. Aven, On the meaning of a black swan in a risk context. *Saf. Sci.* **57**, 44–51 (2013).

15. D. Sharon, H. Kutiel, The distribution of rainfall intensity in Israel, its regional and seasonal variations and its climatological evaluation. *J. Climatol.* **6**, 277–291 (1986).

16. F. Marra, E. Morin, Use of radar QPE for the derivation of Intensity–Duration–Frequency curves in a range of climatic regimes. *J. Hydrol.* **531**, 427–440 (2015).

17. M. Armon, E. Dente, J. A. Smith, Y. Enzel, E. Morin, Synoptic-scale control over modern rainfall and flood patterns in the Levant drylands with implications for past climates. *J. Hydrometeorol.* **19**, 1077–1096 (2018).

18. Y. Rinat, F. Marra, M. Armon, A. Metzger, Y. Levi, P. Khain, E. Vadislavsky, M. Rosensaft, E. Morin, Hydrometeorological analysis and forecasting of a 3 d flash-flood-Triggering desert rainstorm. *Nat. Hazards Earth Syst. Sci.* **21**, 917–939 (2021).

19. S. Tooth, Process, form and change in dryland rivers: A review of recent research. *Earth Sci. Rev.* **51**, 67–107 (2000).

20. R. Serrano-Notivoli, A. Martínez-Salvador, R. García-Lorenzo, D. Espín-Sánchez, C. Conesa-García, Rainfall-runoff relationships at event scale in western Mediterranean ephemeral streams. *Hydrol. Earth Syst. Sci.* **26**, 1243–1260 (2022).

21. E. M. El Khalki, Y. Tramblay, C. Massari, L. Brocca, V. Simonneaux, S. Gascoin, M. E. M. Saidi, Challenges in flood modeling over data-scarce regions: How to exploit globally available soil moisture products to estimate antecedent soil wetness conditions in Morocco. *Nat. Hazards Earth Syst. Sci.* **20**, 2591–2607 (2020).

22. M. Armon, A. J. de Vries, F. Marra, N. Peleg, H. Wernli, Saharan rainfall climatology and its relationship with surface cyclones. *Weather Clim. Extrem.* **43**, 100638 (2024).

23. M. M. El Osta, M. H. Masoud, Implementation of a hydrologic model and GIS for estimating Wadi runoff in Dernah area, Al Jabal Al Akhadar, NE Libya. *J. African Earth Sci.* **107**, 36–56 (2015).

24. J. Jandora, J. Říha, "*The failure of embankment dams due to overtopping*" (Brno Univ. of Technology Vutium Press, 2008).

25. M. Capasso, J. Czerep, A. Dessì, G. Sanchez, Libya Country Report. *EU-LISTCO* (2019); https://eu-listco.net/libya-country-report/.

26. K. He, Q. Yang, X. Shen, E. Dimitriou, A. Mentzafou, C. Papadaki, M. Stoumboudi, E. N. Anagnostou, Brief communication: Storm Daniel Flood Impact in Greece 2023: Mapping crop and livestock exposure from SAR. *Nat. Hazards Earth Syst. Sci.* **24**, 2375–2382 (2024).

27. M. M. Miglietta, R. Rotunno, Development mechanisms for Mediterranean tropical-like cyclones (medicanes). *Q. J. Roy. Meteorol. Soc.* **145**, 1444–1460 (2019).

28. R. Romero, K. Emanuel, Medicane risk in a changing climate. *J. Geophys. Res. Atmos.* **118**, 5992–6001 (2013).

29. L. Aragão, F. Porcù, Cyclonic activity in the Mediterranean region from a high-resolution perspective using ECMWF ERA5 dataset. *Clim. Dyn.* **58**, 1293–1310 (2022).

30. A. Scherrmann, H. Wernli, E. Flaounas, Origin of low-tropospheric potential vorticity in Mediterranean cyclones. *Weather Clim. Dyn.* **4**, 157–173 (2023).

31. M. Sprenger, G. Fragkoulidis, H. Binder, M. Croci-Maspoli, P. Graf, C. M. Grams, P. Knippertz, E. Madonna, S. Schemm, B. Škerlak, H. Wernli, Global climatologies of Eulerian and Lagrangian flow features based on ERA-Interim. *Bull. Am. Meteorol. Soc.* **98**, 1739–1748 (2017).

32. F. Pastor, J. A. Valiente, S. Khodayar, A warming Mediterranean: 38 years of increasing sea surface temperature. *Remote Sens.* **12**, 2687 (2020).

33. F. Marra, D. Zoccatelli, M. Armon, E. Morin, A simplified MEV formulation to model extremes emerging from multiple nonstationary underlying processes. *Adv. Water Resour.* **127**, 280–290 (2019).

34. N. Greenbaum, Y. Enzel, A. P. Schick, Magnitude and frequency of paleofloods and historical floods in the Arava basin, Negev Desert, Israel. *Isr. J. Earth Sci.* **50**, 159–186 (2001).

35. P. Tarolli, M. Borga, E. Morin, G. Delrieu, Analysis of flash flood regimes in the North-Western and South-Eastern Mediterranean regions. *Nat. Hazards Earth Syst. Sci.* **12**, 1255–1265 (2012).

36. Y. Enzel, Y. Kushnir, J. Quade, The middle Holocene climatic records from Arabia: Reassessing lacustrine environments, shift of ITCZ in Arabian Sea, and impacts of the southwest Indian and African monsoons. *Glob. Planet. Change* **129**, 69–91 (2015).

37. J. C. Rieder, F. Aemisegger, E. Dente, M. Armon, Meteorological ingredients of heavy precipitation and subsequent lake filling episodes in the northwestern Sahara. *EGUsphere* **2024**, 1–47 (2024).

38. B. L. Rhoads, Stream power terminology. *Prof. Geogr.* **39**, 189–195 (1987).

39. U.-H. ROAS, Derna damage map (2023); https://unhabitat-roas.hub.arcgis.com/maps/unhabitat-roas::derna-damage-map/about.

40. F. J. Magilligan, Thresholds and the spatial variability of flood power during extreme floods. *Geomorphology* **5**, 373–390 (1992).

41. I. Anderson, D. M. Rizzo, D. R. Huston, M. M. Dewoolkar, Stream power application for bridge-damage probability mapping based on empirical evidence from Tropical Storm Irene. *J. Bridg. Eng.* **22**, 5017001 (2017).

42. A. Ashoor, A. Eladawy, Watch and Upgrade or Deconstruct and Relocate: Derna Catastrophe Lessons Amid the Climate-change Era of Unpredictable Flash Floods. [Preprint (version 1)] (2024). https://doi.org/10.21203/rs.3.rs-3858769/v1.

43. M. Zachariah, V. Kotroni, L. Kostas, C. Barnes, J. Kimutai, S. Kew, I. Pinto, N. Bloemendaal, W. Yang, M. Vahlberg, R. Singh, L. Thalheimer, F. E. L. Otto, Interplay of Climate Change-Exacerbated Rainfall, Exposure and Vulnerability Led to Widespread Impacts in the Mediterranean Region, *World Weather Attribution* (2023).

44. E. Morin, To know what we cannot know: Global mapping of minimal detectable absolute trends in annual precipitation. *Water Resour. Res.* **47**, W07505 (2011).

45. G. Benito, J. A. Ballesteros-Cánovas, A. Díez-Herrero, "Paleoflood hydrology: reconstructing rare events and extreme flood discharges" in *Hydro-Meteorological Hazards, Risks, and Disasters*, J. F. Shroder, P. Paron, G. Di Baldassarre, Eds. (Elsevier, Boston, ed. 2, 2023), vol. 5, pp. 33–83; www.sciencedirect.com/science/article/pii/B9780128191019000091.

46. Z. Kalantari, C. S. S. Ferreira, S. Keesstra, G. Destouni, Nature-based solutions for flood-drought risk mitigation in vulnerable urbanizing parts of East-Africa. *Curr. Opin. Environ. Sci. Health.* **5**, 73–78 (2018).

47. C. S. Ferreira, S. Mourato, M. Kasanin-Grubin, A. J. D. Ferreira, G. Destouni, Z. Kalantari, Effectiveness of nature-based solutions in mitigating flood hazard in a mediterranean peri-urban catchment. *Water* **12**, 2893 (2020).

48. E. Morin, Y. Jacoby, S. Navon, E. Bet-Halachmi, Towards flash-flood prediction in the dry Dead Sea region utilizing radar rainfall information. *Adv. Water Resour.* **32**, 1066–1076 (2009).

49. J. Cools, P. Vanderkimpen, G. El Afandi, A. Abdelkhalek, S. Fockedey, M. El Sammany, G. Abdallah, M. El Bihery, W. Bauwens, M. Huygens, An early warning system for flash floods in hyper-arid Egypt. *Nat. Hazards Earth Syst. Sci.* **12**, 443–457 (2012).

50. H. Hersbach, B. Bell, P. Berrisford, S. Hirahara, A. Horányi, J. Muñoz-Sabater, J. Nicolas, C. Peubey, R. Radu, D. Schepers, A. Simmons, C. Soci, S. Abdalla, X. Abellan, G. Balsamo, P. Bechtold, G. Biavati, J. Bidlot, M. Bonavita, G. De Chiara, P. Dahlgren, D. Dee, M. Diamantakis, R. Dragani, J. Flemming, R. Forbes, M. Fuentes, A. Geer, L. Haimberger, S. Healy, R. J. Hogan, E. Hólm, M. Janisková, S. Keeley, P. Laloyaux, P. Lopez, C. Lupu, G.

Radnoti, P. de Rosnay, I. Rozum, F. Vamborg, S. Villaume, J.-N. Thépaut, The ERA5 global reanalysis. *Q. J. Roy. Meteorol. Soc.* **146**, 1999–2049 (2020).

51. G. J. Huffman, D. T. Bolvin, D. Braithwaite, K. L. Hsu, R. J. Joyce, C. Kidd, E. J. Nelkin, S. Sorooshian, E. F. Stocker, J. Tan, D. B. Wolff, P. Xie, Integrated Multi-satellite Retrievals for the Global Precipitation Measurement (GPM) Mission (IMERG), in *Advances in Global Change Research* (Springer, 2020), vol. 67, pp. 343–353.

52. T. Dinku, P. Ceccato, S. J. Connor, Challenges of satellite rainfall estimation over mountainous and arid parts of east africa. *Int. J. Remote Sens.* **32**, 5965–5979 (2011).

53. J. Tan, G. J. Huffman, D. T. Bolvin, E. J. Nelkin, IMERG V06: Changes to the morphing algorithm. *J. Atmos. Oceanic Tech.* **36**, 2471–2482 (2019).

54. E. Gilleland, D. Ahijevych, B. G. Brown, B. Casati, E. E. Ebert, Intercomparison of spatial forecast verification methods. *Weather Forecast* **24**, 1416–1430 (2009).

55. F. Marra, V. Levizzani, E. Cattani, Changes in extreme daily precipitation over Africa: Insights from a non-asymptotic statistical approach. *J. Hydrol. X.* **16**, 100130 (2022).

56. D. A. Woolhiser, R. E. Smith, D. C. Goodrich, KINEROS: A kinematic runoff and erosion model: Documentation and user manual, *U.S. Department of Agriculture, Agricultural Research Service* (1990).

57. D. C. Goodrich, I. S. Burns, C. L. Unkrich, D. J. Semmens, D. P. Guertin, M. Hernandez, S. Yatheendradas, J. R. Kennedy, L. R. Levick, KINEROS2/AGWA: Model use, calibration, and validation. *Trans. ASABE* **55**, 1561–1574 (2012).

58. O. Z. Al-Hamdan, M. Hernandez, F. B. Pierson, M. A. Nearing, C. J. Williams, J. J. Stone, J. Boll, M. A. Weltz, Rangeland hydrology and erosion model (RHEM) enhancements for applications on disturbed rangelands. *Hydrol. Process.* **29**, 445–457 (2015).

59. H. Wei, M. A. Nearing, J. J. Stone, D. P. Guertin, K. E. Spaeth, F. B. Pierson, M. H. Nichols, C. A. Moffet, A new splash and sheet erosion equation for rangelands. *Soil Sci. Soc. Am. J.* **73**, 1386–1392 (2009).

60. O. Z. Al-Hamdan, F. B. Pierson, M. A. Nearing, C. J. Williams, M. Hernandez, J. Boll, S. K. Nouwakpo, M. A. Weltz, K. Spaeth, Developing a parameterization approach for soil erodibility for the Rangeland Hydrology and Erosion Model (RHEM). *Trans. ASABE* **60**, 85–94 (2017).

61. JAXA/METI, ALOS PALSAR L1.0. (2007). ASF DAAC, https://doi.org/10.5067/J4JVCFDDPEW1 [accessed 20 September 2023].

62. D. R. Montgomery, W. E. Dietrich, Where do channels begin? *Nature* **336**, 232–234 (1988).

63. A. Ashoor, A. Eladawy, Watch and Upgrade or Deconstruct and Relocate: Derna Catastrophe Lessons Amid the Climate-change Era of Unpredictable Flash Floods. [Preprint (version 1)] (2024). https://doi.org/10.21203/rs.3.rs-3858769/v1.

64. T. Hengl, M. A. E. Miller, J. Križan, K. D. Shepherd, A. Sila, M. Kilibarda, O. Antonijević, L. Glušica, A. Dobermann, S. M. Haefele, S. P. McGrath, G. E. Acquah, J. Collinson, L. Parente, M. Sheykhmousa, K. Saito, J. M. Johnson, J. Chamberlin, F. B. T. Silatsa, M. Yemefack, J. Wendt, R. A. MacMillan, I. Wheeler, J. Crouch, African soil properties and nutrients mapped at 30 m spatial resolution using two-scale ensemble machine learning. *Sci. Rep.* **11**, 6130 (2021).

65. T. A. Neumann, A. Brenner, D. Hancock, J. Robbins, A. Gibbons, J. Lee, K. Harbeck, J. Saba, S. B. Luthcke, T. Rebold, ATLAS/ICESat-2 L2A Global Geolocated Photon Data, Version 6. *NASA National Snow and Ice Data Center DistributedActive Archive Center* (2023); https://doi.org/10.5067/ATLAS/ATL03.006.

66. C. Gazzini, When the Dams in Libya Burst: A Natural or Preventable Disaster? (International Crisis Group, 2023).

67. Hydrologic Engineering Center, HECRAS 2D Manual, *US Army Corps of Engineers, Hydrologic Engineering Center* (2023).

68. A. Ashoor, A. Eladawy, Navigating catastrophe: Lessons from Derna amid intensified flash floods in the Anthropocene. *Euro-Mediterranean J. Environ. Integr.* **9**, 1125–1140 (2024).

69. F. Marra, M. Borga, E. Morin, A unified framework for extreme subdaily precipitation frequency analyses based on ordinary events. *Geophys. Res. Lett.* **47**, e90209 (2020).

70. E. Dente, N. G. Lensky, E. Morin, T. Dunne, Y. Enzel, Sinuosity evolution along an incising channel: New insights from the Jordan River response to the Dead Sea level fall. *Earth Surf. Process. Landforms* **44**, 781–795 (2019).

71. J. M. Maurer, J. M. Schaefer, J. B. Russell, S. Rupper, N. Wangdi, A. E. Putnam, N. Young, Seismic observations, numerical modeling, and geomorphic analysis of a glacier lake outburst flood in the Himalayas. *Sci. Adv.* **6**, eaba3645 (2020).

72. J. Quade, E. Dente, A. Cartwright, A. Hudson, S. Jimenez-Rodriguez, D. McGee, Central Andean (28–34 S) flood record 0–25 ka from Salinas del Bebedero, Argentina. *Quatern. Res.* **109**, 207–208 (2022).

73. Y. Guo, Y. Ge, P. Mao, T. Liu, A comprehensive analysis of Holocene extraordinary flood events in the Langxian gorge of the Yarlung Tsangpo River valley. *Sci. Total Environ.* **863**, 160942 (2023).
